# Applications and prospects of different functional hydrogels in meniscus repair

**DOI:** 10.3389/fbioe.2022.1082499

**Published:** 2022-12-08

**Authors:** Pan Jin, Lei Liu, Xichi Chen, Lin Cheng, Weining Zhang, Gang Zhong

**Affiliations:** ^1^ Health Science Center, Yangtze University, Jingzhou, China; ^2^ Collaborative Innovation Centre of Regenerative Medicine and Medical BioResource Development and Application Co-constructed by the Province and Ministry, Guangxi Medical University, Nanning, China; ^3^ Articular Surgery, The Second Nanning People’s Hospital (Third Affiliated Hospital of Guangxi Medical University), Nanning, China; ^4^ Center for Materials Synthetic Biology, CAS Key Laboratory of Quantitative Engineering Biology, Shenzhen Institute of Synthetic Biology, Shenzhen Institutes of Advanced Technology, Chinese Academy of Sciences, Shenzhen, China

**Keywords:** hydrogel, meniscus, function, extracellular matrix, cell, tissue engineering

## Abstract

The meniscus is a kind of fibrous cartilage structure that serves as a cushion in the knee joint to alleviate the mechanical load. It is commonly injured, but it cannot heal spontaneously. Traditional meniscectomy is not currently recommended as this treatment tends to cause osteoarthritis. Due to their good biocompatibility and versatile regulation, hydrogels are emerging biomaterials in tissue engineering. Hydrogels are excellent candidates in meniscus rehabilitation and regeneration because they are fine-tunable, easily modified, and capable of delivering exogenous drugs, cells, proteins, and cytokines. Various hydrogels have been reported to work well in meniscus-damaged animals, but few hydrogels are effective in the clinic, indicating that hydrogels possess many overlooked problems. In this review, we summarize the applications and problems of hydrogels in extrinsic substance delivery, meniscus rehabilitation, and meniscus regeneration. This study will provide theoretical guidance for new therapeutic strategies for meniscus repair.

## Introduction

The meniscus is a semilunar fibro-cartilaginous tissue located in the knee joint that serves as a cushion at the ends of bones. The main component of the meniscus is the extracellular matrix (ECM), which consists of 72% water, 22% collagen, and 0.8% glycosaminoglycans (GAGs) (Tan and Cooper-White, 2011). The remaining components are mostly proteins, glycoproteins, and fibrochondrocytes interspersed in the meniscus. These cells contain a large number of collagen fibers arranged in bundles, which are aligned with the direction of external force to withstand stress, tension, extrusion, and rotary force (Chen S. et al., 2017). Located between the femoral condyle and the tibia, the meniscus is prone to tear when subjected to longitudinal compression force and transverse shear force at the same time. The greater the degree of knee flexion, the more posterior the tear sites. Meniscus tear is the most common meniscus injury, and its severity varies with the location, type, and shape of the tear. According to the blood supply, the meniscus can be divided into a red zone (lateral area with abundant blood supply), a middle zone (central area with less blood supply), and a white zone (medial area with almost no blood supply). The difficulty of healing increases dramatically from the outside to the inside. Meniscus tears come in many forms: longitudinal, horizontal, and radial. If there is no effective treatment, these three tears can develop into bucket handle tear (longitudinal), flap tear (horizontal), or parrot’s beak tear (radial). Meniscus injury, especially white-on-white meniscus tear, lacks regeneration capacity and frequently leads to arthralgia and osteoarthritis. Repair of the meniscus is important for maintaining knee homeostasis and articular surface integrity. Currently, the common repair methods for meniscus injury include meniscectomy, meniscus rehabilitation, and regeneration. Knee joints are more prone to arthritis because meniscectomy destroys the articular surface and joint stability. With the deepening of related research and the increase in quality of life standards, it has become widely accepted that meniscus injury should be repaired rather than excised (Banovetz et al., 2022).

Combining biology and materials science, tissue engineering offers a new approach to meniscus repair. As prominent biomaterials, hydrogels have received much attention for their good biocompatibility, reduction of shear force, and strong plasticity (Longo et al., 2012). They can be used in biomedicine under reasonable structural and functional design (Abazari et al., 2022; Li et al., 2022d). Modification of hydrogels with different responsive materials is a common technique in tissue engineering to optimize the physical and chemical properties of hydrogels (Zhang L. et al., 2022; Zhao P. et al., 2022; Cao et al., 2022; Zhang Q. et al., 2022; Liu et al., 2022; Shao et al., 2022; Shen et al., 2022; Wu et al., 2022). In the published literature, hydrogels have been adopted as scaffolds to deliver exogenous drugs, cells, and factors to promote meniscus rehabilitation and regeneration (Resmi et al., 2020; An et al., 2021; Li et al., 2022b; Zhao Y. P. et al., 2022; Li et al., 2022c). Meanwhile, hydrogel-based repair materials have been fabricated with bioprinting technology and computer three-dimensional (3D) modeling technology to make full use of their good plasticity and printable features (Shi et al., 2021; Gunes et al., 2022; Janarthanan et al., 2022).

In this review, the applications of hydrogels to extrinsic substance-delivery, meniscus rehabilitation, and meniscus regeneration will be illustrated. A comprehensive search of the English articles was conducted in August 2022 with PubMed, Medline and Embase. The search keywords of this review are “hydrogel, mensicus”. No more than 200 articles were found in each database. These articles conatin a variety of literatures including original atricles, review papers, chapters and comments. Duplicate articles are further screened to eliminate them. After the subjective assessment was included, 181 outcomes were identified as relevant to the topic of our interest. Of these, 149 articles were carefully read and made into this review. During the subsequent overhaul, four newly published articles (Baysan G et al., 2022; Herrera Millar et al., 2022; Kim and Bonassar, 2022; Zihna et al., 2022) were added to this review. Next, the various functionalized hydrogels for meniscus repair in the published literature will be overviewed, and the characteristics and applications of these hydrogels will be briefly introduced. This review may illuminate a new direction for the development of hydrogels for meniscus repair in the clinic.

## Extrinsic substance-delivery hydrogels

### Drug-delivery hydrogels

Conventional drug administration methods, including oral and intravenous infusion, frequently result in large fluctuations in blood drug concentrations. Once the concentration falls below the effective concentration, the drug has no effect. On the contrary, if the concentration exceeds the tolerance value, cells and tissues may be damaged. Frequent small dose administration can avoid excessive fluctuation of blood concentration, but it is unlikely to be accepted due to inconvenience. Because hydrogels contain numerous voids and exhibit slow degradation, they are suitable candidates for drug delivery systems (Narayanaswamy and Torchilin, 2019). Good biocompatibility, easily controlled administration, and sustained drug release have enabled hydrogels to play important roles in cancer therapy (Norouzi et al., 2016; Sonker et al., 2021; Xiao et al., 2021). To date, hydrogels have been adopted as drug delivery systems in wound healing (Abazari et al., 2022; Zhao P. et al., 2022; Liu et al., 2022), arthritis therapy (Zhang M. et al., 2022; Zhao Y. P. et al., 2022), ophthalmic disease (Lin et al., 2019; Lynch et al., 2020; Akulo et al., 2022; Das et al., 2022), skin disease (Jiang T. et al., 2018; Yuan et al., 2020), vaginal infections (Perinelli et al., 2018; Dos Santos et al., 2020), and other applications (Jacob et al., 2019). Unfortunately, there have been relatively few studies on the effect of drugs on meniscus repair. Petersen et al. found that the healing effect on non-traumatic meniscus lesions seemed to be equal among surgical and non-surgical treatments (Petersen et al., 2015), but a study by Krych et al. demonstrated that non-operative treatment of medial meniscus posterior horn root tears led to worse clinical outcomes (Krych et al., 2017). Research by Lim et al. revealed that non-operative treatments including non-steroidal anti-inflammatory drugs provided symptomatic relief and functional improvements in most patients with degenerative posterior root tear of the medial meniscus (Lim et al., 2010), and Heo et al. showed that a riboflavin-loaded hydrogel reduced scaffold contraction, increased mechanical properties, and delayed enzyme-triggered degradation of collagen scaffolds (Heo et al., 2016). Zhang et al. reported that simvastatin-conjugated gelatin hydrogel promotes the regeneration of an avascular meniscus in the rabbit model of a meniscal defect (Zhang et al., 2016). Tanaka et al., revealed that intra-articular administration of simvastatin-conjugated gelatin hydrogel attenuates of osteoarthritis progression in mice with down-regulation of autophagic marker and inflammatory factors (Tanaka et al., 2019). Tsubosaka et al., compared the effect of eaicosapentanoic acid (EPA) alone and EPA-incorporating gelatin hydrogels on osteoarthritis (OA) progression with animal investigation in C57BL/6J mice, and found that EPA-incorporating gelatin hydrogels prevent OA progression *in vivo* more effectively than EPA injection alone (Tsubosaka et al., 2020). Wang et al. Reported that Dexamethasone-loaded thermo-sensitive hydrogel exhibits pain-relieving effect in destabilization of medial meniscus (DMM)-induced osteoarthritis of mice models *in vivo* (Wang et al., 2021). Based on the above studies, we preliminarily believe that drugs used in combination with hydrogels can partially improve joint function and alleviate the discomfort symptoms of meniscus injury by down-regulating the inflammatory environment of the joint cavity, but the repair effect of drugs alone on the organic lesions of the meniscus is relatively weak.

### Cell-delivery hydrogels

Hydrogels provide cells with a scaffold that promotes cell proliferation and differentiation while preventing cell loss. Cells can be easily loaded onto hydrogels, resulting in numerous interactions that reproduce the natural interactions of cells and the ECM in tissues. Research by Vernerey et al. suggested that at least five mechanisms, namely communication, mechanosensing, migration, growth, and tissue deposition and elaboration, are involved in cell-hydrogel interactions (Vernerey et al., 2021), which indicates that cells and hydrogels influence each other in tissue engineering applications (Madl and Heilshorn, 2018; Ma and Huang, 2020; Mills et al., 2020). Stem cells are often used for treating human disease because of their multidirectional differentiation potential (Hoang et al., 2022). Cell-loaded hydrogels have been reported to be widely used in tissue engineering. Li et al. suggested that a hydrogel coated with stem cells has a significant effect on the repair of spinal cord injury (Li et al., 2022d). Cell-loaded hydrogels fabricated into either lamellar or 3D forms have been used in treatments for bone and cartilage defects (Liu et al., 2021; Nadine et al., 2022). Due to their high capacity for moisture and good moisturizing effect, porous hydrogels coated with stem cells have been reported to be effective in treating skin wounds (Shafei et al., 2020), limbal stem cell deficiency (Yazdani et al., 2019), damaged kidneys (Jansen et al., 2017), injured cardiac tissue (Waters et al., 2018), and other conditions (Zarrintaj et al., 2021). Meniscal fibrochondrocytes are the main cellular components of the meniscus. Multiple studies have shown that meniscal fibrochondrocytes can form meniscus-like tissue *in vitro* and promote meniscus regeneration *in vivo* (Simson et al., 2013; Chen et al., 2019; Lan et al., 2021). Simson et al. encapsulated bovine meniscal fibrochondrocytes in chondroitin sulfate (CS)-bone marrow (BM) hydrogel (CSBM) hydrogels, and found that meniscal fibrochondrocytes were able to survive, proliferate, and produce meniscus ECM *in vitro*, and meniscus explants adhered by C30B70 fused together after 12 weeks’ implantation in a subcutaneous model of athymic rats (Simson et al., 2013). Baek et al. fabricated a biodegradable and biomimetic nanofibrous scaffold with electrospinning with a biomimetic gel, and demonstrated that cells from avascular and vascular regions of human menisci survived, attached, and infiltrated the scaffold, and secreted the major proteins found in meniscal matrix (Baek et al., 2015). Heo et al. encapsulated fibrochondrocytes isolated from New Zealand white rabbit with Photo-crosslinked collagen-HA hydrogel, and found that gene expression of collagen II and aggrecan was obviously up-regulated (Heo et al., 2016). Chen et al. conducted *in vitro* study and meniscus defect implantation with meniscal fibrochondrocytes (MFCs) and poly (ε-caprolactone) (PCL)-meniscus extracellular matrix (MECM) hydrogel, and revealed that 2% of meniscus extracellular matrix (MECM)-based hydrogel strongly enhanced chondrogenic marker mRNA expression and cell proliferation, and the regenerated menisci in the PCL-hydrogel-MFCs group had similar histological structures, biochemical contents and biomechanical properties as the native menisci in the sham operation group (Chen et al., 2019). Bahcecioglu et al. cultured fibrochondrocytes in agorose, methacrylated gelatin (GelMA), methacrylated hyaluronic acid (MeHA) and GelMA-MeHA blend hydrogels, and found that these hydrogels are more supportive for *in vitro* meniscus regeneration (Bahcecioglu et al., 2019b). Lan et al. mixed human meniscus fibrochondrocytes (hMFC) with 3D bioprinted TEMPO (2,2,6,6-tetramethylpiperidine-1-oxyl)-oxidized cellulose nanofiber-alginate hydrogel and then conducted 6 weeks *in vitro* chondrogenesis’ culture, and found that COL2A1 was highly expressed and more inner meniscus-like phenotype expressed in the TCNF/ALG and collagen-based construct (Lan et al., 2021). Meniscus injury is common, but the number of meniscus cells (MCs) that can be recycled is limited. Kremer et al. compared primary equine mesenchymal stem cells (MSCs) and MCs on three different scaffolds and found that the phenotype of MSCs and MCs co-cultured on a scaffold composed of Col I gel on SIS-muc exhibited the greatest similarity to native meniscus tissue (Kremer et al., 2017). Hagmeijer determined that 20% MCs and 80% MSCs were the most appropriate ratio for a type I collagen hydrogel for meniscus regeneration, and the stimulatory effect of MSCs towards meniscus cells was demonstrated by cellular communication through gap junctions (Hagmeijer et al., 2019). Co-culture of stem cells with MCs is beneficial for stem cells to differentiate into fibrochondrocytes. If MCs could be replaced with stem cells, it would facilitate the continued development of meniscus tissue engineering. Koh et al. encapsulated conditioned medium (CM)-expanded human tonsil-derived mesenchymal stem cells (T-MSCs) in riboflavin-induced photocrosslinked collagen-hyaluronic acid (COL-RF-HA) hydrogels, and cultured in chondrogenic medium containing TGF-β3 *in vitro* and implanted subcutaneously in female nude Balb-c mice *in vivo*, and he found that CM-expanded cells support highest cell proliferation, GAG accumulation, and collagen deposition and CM treatment induced complete regeneration when implanted into meniscus defect model (Koh et al., 2017). Yuan et al. firstly buried mECM hydrogel encapsulated with human mesenchymal stem cells (hMSCs) under the skin and implanted subcutaneously for an additional 4 weeks, and then the *in situ* model of meniscal injury was conducted in orthotopic model of meniscal injury in nude rat. After these procedures, he revealed that decellularized meniscus ECM hydrogel retained tissue-specific proteoglycans and collagens, and significantly upregulated expression of fibrochondrogenic markers, and the meniscus ECM hydrogel in turn supported delivery of hMSCs for integrative repair of a full-thickness defect model in meniscal explants after *in vitro* culture and *in vivo* subcutaneous implantation (Yuan et al., 2017). A research of Romanazzo et al. suggested that inner meniscus ECM promoted chondrogenesis of fat pad-derived stem cells with exogenous growth factors, and inner ECM-functionalised hydrogels supported the highest levels of Sox-9 and type II collagen gene expression and sulfated glycosaminoglycans (sGAG) deposition when supplemented with TGFβ3. Whereas, outer meniscus ECM promoted a more elongated cell morphology and the development of a more fibroblastic phenotype In the absence of exogenously supplied growth factors, and a more fibrogenic phenotype was observed in outer ECM-functionalised hydrogels supplemented with connective tissue growth factor (Romanazzo et al., 2018). Chen et al. encapsulated bone mesenchymal stromal cells into A thermosensitive, injectable, *in situ* crosslinked hydrogel, and found that hydrogel was biocompatible and could stimulate strong fibrochondrogenic differentiation of BMSCs after the incorporation of TGF-β1, and local administration of the BMSC-laden, TGF-β1-incorporated hydrogel could promote the healing of rabbit meniscal injury (Chen et al., 2020). Zhong et al. investigated the effect of mECM on encapsulated MSCs and integrative meniscus repair by *in vivo* rat subcutaneous implantation and orthotopic meniscus injury model, and revealed that BMSCs-laden mECM hydrogels promote meniscus regeneration and improve joint function (Zhong et al., 2020). These studies suggest that cell-loaded hydrogels can be a promising strategy for meniscus repair. Although both MCs and stem cells have been reported to be useful in meniscus repair, more research is needed to compare their repair effects and underlying mechanisms.

### Protein and cytokine-delivery hydrogels

**FIGURE 1:**
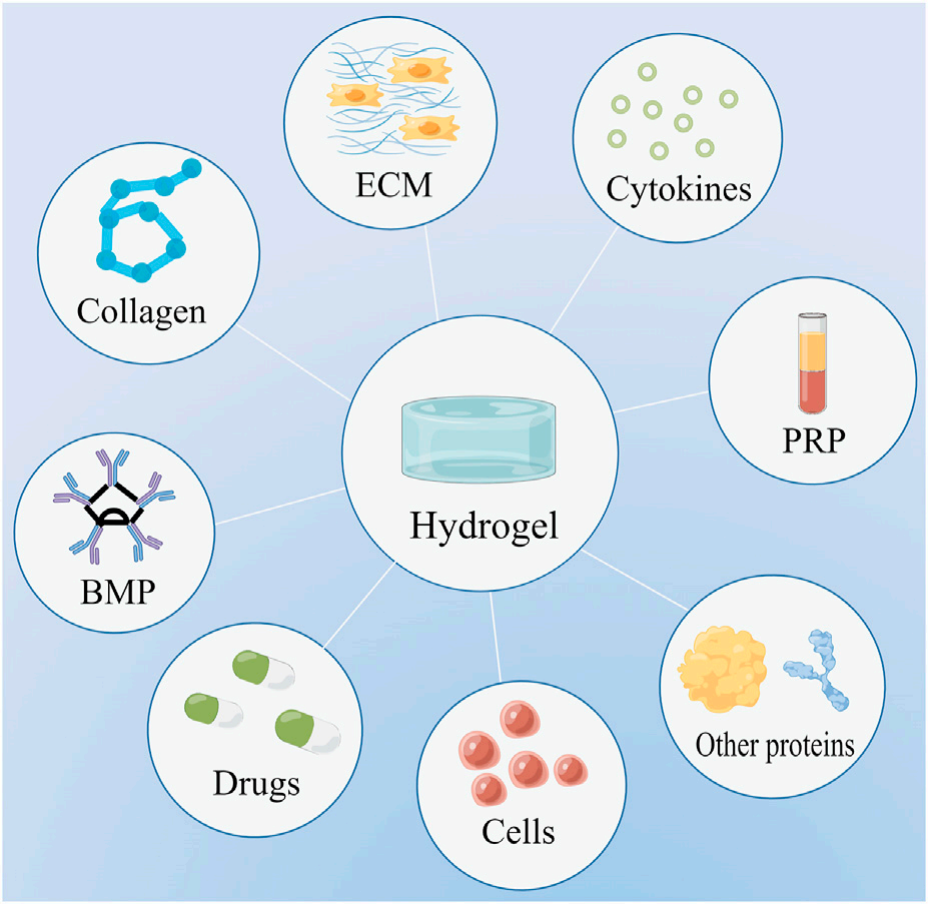
Hydrogels can serve as scaffolds to deliver a variety of substances, including drugs, cells, extracellular matrix (ECM), bone morphogenetic protein (BMP), collagen, cytokines, platelet-rich plasma (PRP), and other proteins.

Because of the interactions between a hydrogel and its loaded cells, the composition and characteristics of the hydrogel directly affect cell fate (Tsou et al., 2016). Delivery of many proteins and cytokines—including bone morphogenetic protein (BMP), transforming growth factor-beta (TGF-β), silk fibroin, and platelet-rich plasma (PRP)—by loading hydrogels with exosomes is reported to be beneficial to the treatment of bone, cartilage, skin, and spinal cord injuries (Qiu et al., 2016; Maisani et al., 2018; Wang L. et al., 2020; Shafei et al., 2020; Cheng et al., 2021; Jiang et al., 2021; Li et al., 2021; Lin et al., 2021; Saygili et al., 2021; Yuan et al., 2021; Zhang et al., 2021; Yan et al., 2022). The ECM is the main extracellular component of the meniscus, and it plays a key role in soft tissue regeneration, providing cells with a dynamic and complex array of biochemical and biomechanical signals that regulate cell adhesion, proliferation, differentiation, and migration (Wu et al., 2021). ECM-functionalized hydrogels are reported to be prominent in enabling meniscus repair when combined with different cells in multiple studies (Baek et al., 2015; Wu et al., 2015; Yuan et al., 2017; Chen et al., 2019; Zhong et al., 2020). Shimomura et al. analyzed the expression of meniscus-associated genes with human bone marrow MSCs (hBMSCs) seeded on inner and outer meniscus-derived ECMs (mECMs) and concluded that ECMs derived from different regions had different effects on the differentiation of stem cells based on the results that inner mECM seeding enhanced the fibrocartilaginous differentiation of hBMSCs, whereas outer mECM seeding promoted a more fibroblastic phenotype (Shimomura et al., 2017). The aforementioned studies indicate that different regions of the meniscus release different factors and substances that stimulate cells to differentiate into cells with different functions, but more research is needed to reveal the underlying mechanisms. Okuno et al. reported that KI24RGDS peptide hydrogel facilitated meniscus repair in a rabbit meniscal defect model (Okuno et al., 2021). *In vitro* and *in vivo* studies by Forriol et al. and Ozeki et al. demonstrated that bone morphogenetic protein-7 (BMP-7) is a suitable growth factor to stimulate meniscus regeneration and delay cartilage degeneration (Ozeki et al., 2013; Forriol et al., 2014). As major components of the ECM, collagen types I, II, and III were all revealed to be key regulators in meniscus repair (Mueller et al., 1999; Oda et al., 2015; Wang C. et al., 2020). In addition, Chen et al. and Narita et al. suggested that transforming growth factor β1 and fibroblast growth factor 2 incorporated with hydrogels enhance the healing effect on meniscus injury (Narita et al., 2012; Chen et al., 2020). Pan et al. demonstrated that conditional EGFR deletion in mice and intra-articular injection of a small molecule EGFR inhibitor gefitinib together could promote ECM production (Pan et al., 2017). Meanwhile, Liang et al. found that transforming growth factor beta-3 (TGF-β3) and insulin-like growth factor 1 (IGF-1) are indispensable growth factors by comparing the effects of these factors on the cell differentiation of synovial fluid-derived MSCs (SF-MSCs) toward meniscus fibrochondrocytes (Liang et al., 2018). Ishida et al. proposed that PRP-laden hydrogel may exert a regenerative effect on the meniscus *in vitro* and *in vivo* (Ishida et al., 2007). Another protein mainly expressed in red blood cells, erythropoietin, was demonstrated to enhance meniscus repair and prevent osteoarthritis formation (Fu et al., 2020). Hao et al. encapsulated chemokines (platelet-derived growth factor-BB, PDGF-BB) and small chondroinductive molecules (kartogenin, KGN) within biomimetic polycaprolactone/hydrogel, and found that the dual drug-releasing meniscal scaffold possesses the potential to act as an off-the-shelf product for the clinical treatment of meniscal injury and related joint degenerative diseases (Hao et al., 2021). The changes and mechanisms of related regulatory factors during meniscus development and following injury have not been clarified, so more research in this area is required to reveal the roles of related proteins and cytokines at different stages. Figure 1 and Table 1 show the representative reseaches of extrinsic substance-delivery hydrogels in meniscus repair and their outcomes.

**TABLE 1:** Summary table of extrinsic substance-delivery hydrogels in meniscus repair and their outcomes.

Classification	Substances that delivered	Hydrogel	Delivery method	Outcomes	References
Drug-delivery hydrogels	Riboflavin	Photo-crosslinked collagen-HA hydrogel	Encapsulated with fibrochondrocytes isolated from New Zealand white rabbit, *in vitro* cell study	Reduced scaffold contraction and enhanced gene expression levels for the collagen II and aggrecan	Heo et al. (2016)
	Simvastatin	Gelatin hydrogel	Implantated into the mensicus defect of Japanese White rabbits, *in vivo* animal experiment	Promoted the regeneration of an avascular meniscus in the rabbit model of a meniscal defect	Zhang et al. (2016)
	Simvastatin	Gelatin hydrogel	Murine primary chondrocytes, *in vitro* cell study; Intra-articular injection in C57BL/6J mice, *in vivo* animal experiment	Down-regulated the expression of inflammatory factors, MMP-13, and autophagic marker LC3	Tanaka et al. (2019)
	Eicosapentanoic acid	Gelatin hydrogel	Intra-articular injection in C57BL/6J mice, *in vivo* animal experiment	Inhibited the expression of macrophages and inflammatory factors, but not mentioned the effect on the meniscus	Tsubosaka et al. (2020)
	Dexamethasone	Thermo-sensitive hydrogel	Intra-articular injection in C57BL/6J mice, *in vivo* animal experiment	Reduced the expression of inflammatory factors, relieves pain and attenuates bone destruction, but not mentions the effect on the meniscus	Wang et al. (2021)
Cell-delivery hydrogels	Bovine meniscal fibrochondrocytes	Chondroitin sulfate (CS)-bone marrow (BM) hydrogel	Encapsulated meniscal fibrochondrocytes in CSBM hydrogels, *in vitro* cell study and subcutaneous implantation in athymic rats	Meniscal fibrochondrocytes were able to survive, proliferate, and produce meniscus ECM when encapsulated in CS-BM hydrogel. In a subcutaneous model to assess meniscus fusion, meniscus explants adhered by C30B70 fused together 12 weeks postimplantation	Simson et al. (2013)
	Human meniscus cells	Extracellular matrix hydrogel	Combined nanofibrous scaffolds with human meniscus cells in an ECM hydrogel, *in vitro* cell study	Supported meniscus tissue formation with increased COL1A1, SOX9, COMP	Baek et al. (2015)
	Rabbit meniscal fibrochondrocytes	Photo-crosslinked collagen-HA hydrogel	Encapsulated rabbit meniscal fibrochondrocytes in the hydrogel, *in vitro* cell study	Beneficial to gene expression of collagen II and aggrecan	Heo et al. (2016)
	Rabbit meniscal fibrochondrocytes (MFCs)	Meniscus extracellular matrix (MECM)-based hydrogel	A 3D printing wedge-shaped poly (ε-caprolactone) (PCL) scaffold as a backbone, *in vitro* cell study and scaffolds were implanted in the meniscus defect and sutured to the residual rim after the mature New Zealand White rabbits underwent total medial meniscectomy except 5% of the external rim *in vivo*	2% of meniscus extracellular matrix (MECM)-based hydrogel strongly enhanced chondrogenic marker mRNA expression and cell proliferation; PCL-hydrogel-MFCs group exhibited markedly better gross appearance and cartilage protection than other groups and the regenerated menisci in the PCL-hydrogel-MFCs group had similar histological structures, biochemical contents and biomechanical properties as the native menisci in the sham operation group	Chen et al. (2019)
	Porcine meniscal fibrochondrocytes	Agorose, methacrylated gelatin (GelMA), methacrylated hyaluronic acid (MeHA) and GelMA-MeHA blend hydrogels	Fibrochondrocytes (passage 2) were reconstituted in the polymer solutions prior to photocuring and then cultured in fibrochondrogenic medium	Hydrogels have a higher potential for meniscal regeneration than the 3D printed PCL, and fibrochondrocytes could be directed to proliferate or produce cartilaginous or fibrocartilaginous ECM. Agarose and MeHA could be used for the regeneration of the inner region of meniscus, while GelMA for the outer region	Bahcecioglu et al. (2019b)
	Human meniscus fibrochondrocytes (hMFC)	TEMPO (2,2,6,6-tetramethylpiperidine-1-oxyl)-oxidized cellulose nanofiber-alginate hydrogel	hMFCs were mixed with TCNF/ALG precursors with suitable formulations and 3D bioprinted into cylindrical disc constructs and crosslinked with CaCl2 after printing. The bioprinted constructs then underwent 6 weeks of *in vitro* chondrogenesis in hypoxia prior to analysis with biomechanical, biochemical, molecular, and histological assays	The TCNF/ALG and collagen-based constructs had similar compression modul and significantly higher expression of COL2A1. Was significantly higher in TCNF/ALG. The TCNF/ALG constructs showed more of an inner meniscus-like phenotype while the collagen I-based construct was consistent with a more outer meniscus-like phenotype	Lan et al. (2021)
	Equine meniscus cells (MCs) and mesenchymal stem cells (MSCs)	Type I collagen hydrogel	Primary equine mesenchymal stem cells (MSC) and meniscus cells (MC) seeded on three different scaffolds-a cell-laden collagen type I hydrogel (Col I gel), a tissue-derived small intestinal matrix scaffold (SIS-muc) and a combination thereof	The phenotype of MSCs and MCs co-cultured on a scaffold composed of Col I gel on SIS-muc exhibited the greatest similarity to native meniscus tissue	Kremer et al. (2017)
	Human meniscus cells (MCs) and mesenchymal stem cells (MSCs)	Type I collagen hydrogel	Cells were seeded on the implant in fibrin glue by static seeding or injection	20% MCs and 80% MSCs were the most appropriate ratio for a type I collagen hydrogel for meniscus regeneration	Hagmeijer et al. (2019)
	Human tonsil-derived mesenchymal stem cells (T-MSCs)	Riboflavin-induced photocrosslinked collagen-hyaluronic acid (COL-RF-HA) hydrogel	Conditioned medium (CM)-expanded T-MSCs were encapsulated in riboflavin-induced photocrosslinked collagen-hyaluronic acid (COL-RF-HA) hydrogels, and cultured in chondrogenic medium containing TGF-β3 *in vitro* and subcutaneously implanted in female nude Balb-c mice *in vivo*	*In vitro* results indicate that CM-expanded cells followed by TGF-β3 exposure stimulated the expression of fibrocartilage-related genes (COL2, SOX9, ACAN, COL1) and production of extracellular matrix components, and CM treatment amplified the potential of TGF-β3 and induced complete regeneration when implanted into meniscus defect model	Koh et al. (2017)
	Human mesenchymal stem cells (hMSCs)	Decellularized juvenile bovine meniscus ECM hydrogel	hHMSCs encapsulated in mECM hydrogel and cultured *in vitro*. After 42 days *in vitro*, hMSC-mECM constructs were implanted subcutaneously for an additional 4 weeks.Then the *in situ* model of meniscal injury was conducted in orthotopic model of meniscal injury in nude rat *in vivo*	Fibrochondrogenesis of hMSCs was superior in mECM hydrogel compared to type I collagen alone. HMSCs in mECM hydrogel enhanced integrative repair of meniscal explants. HMSCs delivered in mECM into meniscal injury incorporated into host tissue	Yuan et al. (2017)
	Porcine infrapatellar fat pad-derived stem cells	Extracellular matrix (ECM)-functionalised hydrogel	Alginate hydrogels were functionalised with ECM derived from the inner and outer regions of the meniscus and loaded with infrapatellar fat pad-derived stem cells. Encapsulation and *in vitro* culture of FPSC in alginate-ECM hydrogels	In the absence of exogenously supplied growth factors, inner meniscus ECM promoted chondrogenesis of fat pad-derived stem cells, whereas outer meniscus ECM promoted a more elongated cell morphology and the development of a more fibroblastic phenotype. With exogenous growth factors supplementation, a more fibrogenic phenotype was observed in outer ECM-functionalised hydrogels supplemented with connective tissue growth factor, whereas inner ECM-functionalised hydrogels supplemented with TGFβ3 supported the highest levels of Sox-9 and type II collagen gene expression and sulfated glycosaminoglycans (sGAG) deposition	Romanazzo et al. (2018)
	Rabbit bone marrow stem cells (BMSCs)	A thermosensitive, injectable, *in situ* crosslinked hydrogel	BMSCs were isolated and cultured in the hydrogel *in vitro*. A critical-sized defect was introduced into the meniscus of 30 rabbits. Each defect was randomly assigned to be implanted with either phosphate-buffered saline (PBS); BMSC-laden hydrogel; or BMSC-laden, TGF-β1-incorporated hydrogel in the *in vivo* experiment	The hydrogel was biocompatible and could stimulate strong fibrochondrogenic differentiation of BMSCs after the incorporation of TGF-β1. The local administration of the BMSC-laden, TGF-β1-incorporated hydrogel could promote the healing of rabbit meniscal injury	Chen et al. (2020)
	Rat bone marrow stem cells (BMSCs)	Decellularized meniscus extracellular matrix (mECM) hydrogel	Encapsulation of BMSCs in mECM or hydrogel and *in vitro* culture; *In vivo* subcutaneous implantation model in SD rat; Orthotopic model of meniscal injury in SD rats, BMSCs encapsulated in mECM or collagen were injected into the defect by 25-gauge needle in the *in vivo* study	Decellularized mECM retained essential proteoglycans and collagens, and significantly upregulated expression of fibrochondrogenic markers by BMSCs *versus* collagen hydrogel alone *in vitro* 3D cell culture. When applied to an orthotopic model of meniscal injury in SD rat, mECM is superior than collagen I scaffold in reduction of osteophyte formation and prevention of joint space narrowing and osteoarthritis development as evidenced by histology and micro-CT analysis	Zhong et al. (2020)
Protein and cytokine-delivery hydrogels	Platelet-rich plasma (PRP)	Gelatin hydrogel (GH)	Monolayer meniscal cell cultures were performed to assess proliferative behavior in the presence of PRP *in vitro*, and 1.5-mm-diameter full-thickness defects were created in the avascular region of rabbit meniscus and implanted with GH with PRP, GH with platelet-poor plasma, or GH only *in vivo*	Histological scoring of the defect sites at 12 weeks revealed significantly better meniscal repair in animals that received PRP with GH than in the other two groups, which suggested that PRP enhances the healing of meniscal defects	Ishida et al. (2007)
	Fibroblast growth factor 2 (FGF-2)	Gelatin hydrogel	The purpose of this study was to investigate the *in vivo* effects of gelatin hydrogels (GHs) incorporating fibroblast growth factor 2 (FGF-2) on meniscus repair in a rabbit model	GHs incorporating FGF-2 significantly stimulated proliferation and inhibited the death of meniscal cells until 4 weeks, thereby increasing meniscal cell density and enhancing meniscal repair in a rabbit model	Narita et al. (2012)
	Collagen type II	A hydrogel consisting of collagen type II (3 mg/ml), chondroitin sulfate (1 mg/ml) and hyaluronan (1 mg/ml)	Human meniscus cells were embedded in extracellular matrix (ECM) hydrogel to lead to formation of neotissues that resemble meniscus-like tissuel, *in vitro* cell study	Supported neotissue formation with high expression of meniscus-related genes	Baek et al. (2015)
	Extracellular matrix (ECM)	An injectable ECM hydrogel material from porcine meniscus	Bovine chondrocytes and mouse 3T3 fibroblasts was encapsulated in the hydrogel, *in vitro* study; syringe injection into mouse subcutaneous tissue, *in vivo* mouse subcutaneous implantation	The *in vitro* study demonstrated that the hydrogel have good cellular compatibility, and the *in vivo* study revealed that the ECM hydrogel possessed good tissue compatibility	Wu et al. (2015)
	Meniscus extracellular matrix (MECM)	Meniscus extracellular matrix (MECM)-based hydrogel	Human bone marrow MSCs (hBMSCs) were seeded on inner and outer meniscus-derived ECMs (mECMs), *in vitro* study	ECMs derived from different regions had different effects. Inner mECM seeding enhanced the fibrocartilaginous differentiation of hBMSCs, whereas outer mECM seeding promoted a more fibroblastic phenotype	Shimomura et al. (2017)
	Meniscus extracellular matrix (MECM)	Decellularized juvenile bovine meniscus ECM hydrogel	Human mesenchymal stem cells (hMSCs) encapsulated in mECM hydrogel and cultured *in vitro*. After 42 days *in vitro*, hMSC-mECM constructs were implanted subcutaneously for an additional 4 weeks.Then the *in situ* model of meniscal injury was conducted in orthotopic model of meniscal injury in nude rat *in vivo*	Fibrochondrogenesis of hMSCs was superior in mECM hydrogel compared to type I collagen alone. HMSCs in mECM hydrogel enhanced integrative repair of meniscal explants. HMSCs delivered in mECM into meniscal injury incorporated into host tissue	Yuan et al. (2017)
	Meniscus extracellular matrix (MECM)	Meniscus extracellular matrix (MECM)-based hydrogel	Rabbit meniscal fibrochondrocytes (MFCs) were seeded in the hydrogel, *in vitro* cell study; PCL-hydrogel-MFCs were implanted in the meniscus defect, *in vivo* rabbit experiment	Both *in vitro* and *in vivo* study revealed that PCL-hydrogel-MFCs benefit to meniscus regeneration	Chen et al. (2019)
	Transforming growth factor β1 (TGF-β1)	A thermosensitive, injectable, *in situ* crosslinked hydrogel	BMSCs were isolated and cultured in the hydrogel *in vitro*, and TGF-β1-incorporated hydrogel was implanted incritical-sized defects *in vivo*	The hydrogel was biocompatible and could stimulate strong fibrochondrogenic differentiation of BMSCs after the incorporation of TGF-β1. The local administration of the BMSC-laden, TGF-β1-incorporated hydrogel could promote the healing of rabbit meniscal injury	Chen et al. (2020)
	Meniscus extracellular matrix (MECM)	Decellularized meniscus extracellular matrix (mECM) hydrogel	Encapsulation of BMSCs in mECM or hydrogel and *in vitro* culture; *In vivo* subcutaneous implantation model in SD rat; Orthotopic model of meniscal injury in SD rat, BMSCs encapsulated in mECM or collagen were injected into the defect by 25-gauge needle in the *in vivo* study	Decellularized mECM retained essential proteoglycans and collagens, and significantly upregulated expression of fibrochondrogenic markers by BMSCs *versus* collagen hydrogel alone *in vitro* 3D cell culture. When applied to an orthotopic model of meniscal injury in SD rat, mECM is superior than collagen I scaffold in reduction of osteophyte formation and prevention of joint space narrowing and osteoarthritis development as evidenced by histology and micro-CT analysis	Zhong et al. (2020)
	Chemokines (platelet-derived growth factor-BB, PDGF-BB) and small chondroinductive molecules (kartogenin, KGN)	Polycaprolactone hydrogel	The scaffold morphology, drug release and the effects of releasing the drugs in a sequentially controlled manner from the composite scaffolds on the fate of MSCs were evaluated, and the healing effect of the hydrogel was assessed in a rabbit model established with a critical-size medial meniscectomy *in vivo*	The meniscal scaffolds containing both drugs had combinational advantages in enhancing cell migration and synergistically promoted MSC chondrogenic differentiation. The dual drug-loaded scaffolds also significantly promoted *in vivo* neomeniscal regeneration three and 6 months after implantation in terms of histological and immunological phenotypes	Hao et al. (2021)
	Peptide	KI24RGDS peptide hydrogel	Full-thickness (2.0 mm diameter) cylindrical defects were introduced into the inner avascular zones of the anterior portions of the medial menisci of rabbit knees *in vivo*	KI24RGDS remained in the meniscal lesion and facilitated the repair and regeneration in a rabbit meniscal defect model	Okuno et al. (2021)

## Meniscus rehabilitation hydrogels

### Biological tissue-derived hydrogels

Conservative treatment and meniscus suture are the traditional meniscus repair methods, but their long-term results are not ideal (Chambers and Chambers, 2019; Ulku et al., 2020). Hydrogels may be viable options in meniscus repair since they are lubricating and injectable. A juvenile bovine menisci-derived ECM hydrogel was proposed to exert therapeutic effects on rat meniscus injury (Yuan et al., 2017). Studies by both Zhong et al. and Ruprecht et al. indicated that porcine meniscus-derived matrix (MDM) hydrogels promote the repair of meniscus injury (Ruprecht et al., 2019; Zhong et al., 2020). Visser et al. processed different kinds of hydrogels with equine cartilage, meniscus, and tendon tissue, and found that cell differentiation can be influenced by different hydrogels (Visser et al., 2015). Meanwhile, Scotti et al. demonstrated that cellular fibrin glue has promising potential in enhancing the meniscus bonding effect (Scotti et al., 2009). Recent research suggests that the endostatin in fibrin hydrogel may be the key element to promoting chondrogenic differentiation of swne neonatal meniscal cells (Herrera Millar et al., 2022). In addition, PRP, which contains multiple proteins and growth factors, was reported to be effective in meniscus healing (Braun et al., 2015). Through a 3-month clinical follow-up trial, Popescu et al. found that PRP played a positive role in adolescents with meniscus lesions (Popescu et al., 2020). On the contrary, studies by Dai et al. and Yang et al. reported that a similar effect in functional outcome and pain relief was found in the PRP group and non-PRP group (Dai et al., 2019; Yang et al., 2021). Considering these contradictory results, we speculate that the complex components in PRP may be the cause. More studies are needed to isolate PRP components and examine the effects of various components.

### Natural biomaterial-derived hydrogels

Raw materials from biological sources are limited and may contain yet undetectable adverse immune effects, so it is necessary to develop hydrogels from natural biomaterials. As a natural polysaccharide, sodium alginate has been widely used in tissue engineering research (Rastogi and Kandasubramanian, 2019). Lan et al. fabricated TEMPO (2,2,6,6-tetramethylpiperidine-1-oxyl)-oxidized cellulose nanofiber/alginate (TCNF/ALG) hydrogel and suggested that human meniscus fibrochondrocytes (hMFC) implanted in the hydrogel exhibited a more inner meniscus-like phenotype, whereas cells cultured in a collagen I-based construct exhibited a more outer meniscus-like phenotype (Lan et al., 2021). As another common hydrogel ingredient, agarose is widely used to prepare gels for separating macromolecular proteins and DNA. Gunja et al. employed agarose molds to detect the effects of agarose mold compliance and surface roughness on self-assembled meniscus-shaped constructs, and they found that 1% agarose exhibited higher potential for preventing construct buckling (Gunja et al., 2009). Hyaluronic acid, a natural moisturizing factor, is mainly used in cosmetology, ophthalmology, and arthrology. Research by Berton et al. and Kim et al. demonstrated that hyaluronic acid hydrogels have a restorative effect in meniscus injury (Kim et al., 2018; Berton et al., 2020). Due to its easy degradation and lack of fixed molecular weight, gelatin is often mixed with other substances (e.g., alginate, agarose, hyaluronic acid, and methacrylate) to fabricate hydrogels (Echave et al., 2017; Salahuddin et al., 2021). A study by Resmi et al. revealed that the injectable alginate dialdehyde-gelatin (15ADA20G) hydrogel shows good integration with the host meniscus tissue and relatively long retention in the close region of meniscus tear (Resmi et al., 2020). Adjusting the PH of gelatin has been reported as a viable method to improve both biochemical and biomechanical properties of hydrogels for tissue-engineered meniscus (Kim and Bonassar, 2022). The main type of collagen in the ECM of the meniscus is type I collagen. An *in vitro* study by Kremer et al. indicated that type I collagen hydrogel is beneficial to the high expression of meniscus-related proteins in stem cells (Kremer et al., 2017).

### Artificial hydrogels

**FIGURE 2:**
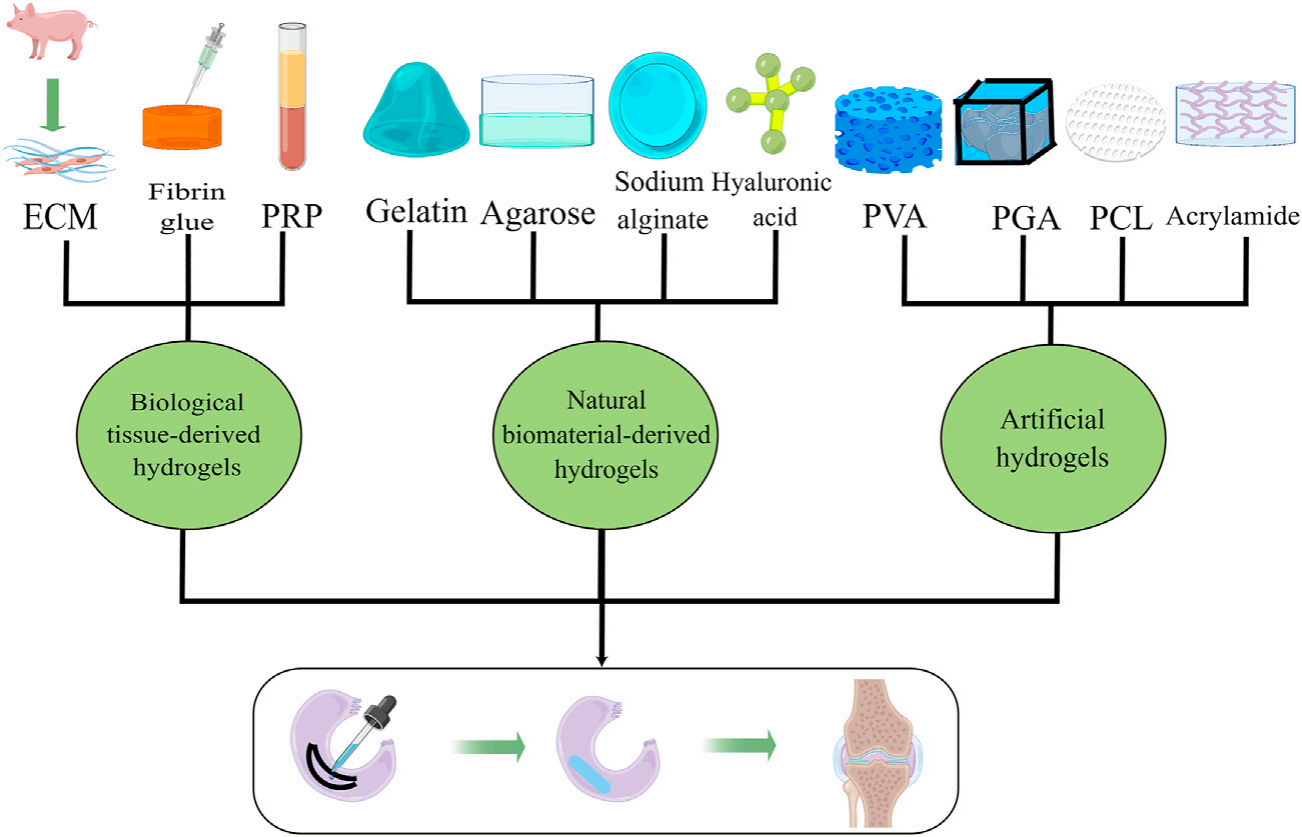
Multiple hydrogels used as repair materials for meniscus rehabilitation, including biological tissue-derived hydrogels, natural biomaterial-derived hydrogels, and artificial hydrogels.

Although natural hydrogels have good biodegradability, artificial hydrogels have better mechanical properties. Acrylamide, a commonly used material for hydrogel preparation, is often used with other natural hydrogel materials or synthetic hydrogel materials. A study by Bahcecioglu et al. stated that gelatin methacrylate (GelMA)-agarose (Ag) hydrogel was suitable for medial meniscus preparation, and GelMA was conducive to lateral meniscus preparation (Bahcecioglu et al., 2019a). Zihna et al. prepared hybrid meniscal constructs using methacrylate gelatin (GelMA) hydrogels and acellular matrices and proved it may be used in meniscus tissue engineering with mechanical tests and *in vitro* cell experiments (Zihna et al., 2022). Modified polyvinyl alcohol (PVA) hydrogel has better load-bearing capacity and lower cytotoxicity than PVA alone, which is suggested to be beneficial to meniscus repair either alone or combined with gelatin (Hayes et al., 2016; Marrella et al., 2018). Zhang et al. fabricated a biodegradable poly (l-glutamic acid) (PLGA)-g-poly (ε-caprolactone) (PCL) hydrogel and proved that the hydrogel carrying adipose-derived stem cells (ASCs) effectively regenerated meniscus-like tissue *in vivo* and preserved the corresponding articular cartilage from degeneration over a 16 week period (Zhang K. et al., 2018). In addition, polycaprolactone (PCL) and poly (glycolic acid) (PGA) hydrogels have been demonstrated to be conducive scaffolds for meniscus repair (Aufderheide and Athanasiou, 2015; Chen et al., 2019). Compared with meniscus repair materials, the current research is more inclined to manufacture integrated materials for restoration and regeneration. Recent study of Baysan et al. revealed that a new type of hydrogel composite scaffold made of chitosan, loofah mat, and poly (-3-hydroxybutyrate-co-3-hydroxyvalerate) (PHBV) nanofibers is a promising material for engineering meniscus tissue (Baysan et al., 2022). Figure 2 and Table 2 present the typical investigations of meniscus rehabilitation hydrogels in meniscus repair and their outcomes.

**TABLE 2:** Summary table of meniscus rehabilitation hydrogels in meniscus repair and their outcomes.

Classification	Hydrogel	Procedures	Outcomes	References
Biological tissue-derived hydrogels	Cellular fibrin glue	The bonding capacity of an articular chondrocytes-fibrin glue hydrogel was tested as a biologic glue to improve the bonding between two swine meniscal slices in a nude mouse model	A fibrocartilaginous tissue was found at the interface between the meniscal slices, partially penetrating the native meniscus tissue	Scotti et al. (2009)
	Bovine meniscus extracellular matrix (ECM) hydrogel	Human mesenchymal stem cells (hMSCs) wew cultured in the meniscus ECM hydrogel *in vitro*, and applied to an orthotopic model of meniscal injury in nude rat *in vivo*	The meniscus ECM hydrogel supported delivery of hMSCs for integrative repair of a full-thickness defect model in meniscal explants	Yuan et al. (2017)
	Decellularized meniscus extracellular matrix (mECM) hydrogel	Rat bone marrow stem cells (BMSCs) were cultured in the hydrogel *in vitro*, and injected into the meniscus defect in orthotopic model of meniscal injury in SD rats *in vivo*	The hydrogel upregulated expression of fibrochondrogenic markers, reduced the osteophyte formation, and prevented joint space narrowing and osteoarthritis development	Zhong et al. (2020)
	Fibrin hydrogel	Swine neonatal meniscal cells were cultured in fibrin hydrogel scaffolds, and endostatin was evaluated to assess the differentiation of avascular tissues	Endostatin in 3D fibrin hydrogel scaffolds promotes chondrogenic differentiation in swine neonatal meniscal cells	Herrera Millar et al. (2022)
Natural biomaterial-derived hydrogels	Agarose	Co-cultures of ACs and MCs (50:50 ratio) were cultured in smooth or rough moulds composed of 1% or 2% agarose for 4 weeks	The topology of an agarose surface may be able to affect the phenotypic properties of cells that are on that surface, with smooth surfaces supporting a more chondrocytic phenotype	Gunja et al. (2009)
	Type I collagen hydrogel	Primary equine mesenchymal stem cells (MSC) and meniscus cells (MC) seeded on three different scaffolds-a cell-laden collagen type I hydrogel (Col I gel), a tissue-derived small intestinal matrix scaffold (SIS-muc) and a combination thereof-for	Type I collagen hydrogel is beneficial to the high expression of meniscus-related proteins in stem cells	Kremer et al. (2017)
	Enzyme-mediated tissue adhesive hydrogels	Rabbit meniscus fibrochondrocytes were cultured in the hydrogel and ECM synthesis and gene expression were detected *in vitro*	Fibrochondrocyte-laden and TYR-crosslinked hydrogels demonstrated strong biocompatibility and resulted in enhancement of cartilage-specific gene expression and matrix synthesis	Kim et al. (2018)
	Hyaluronic acid (HA) hydrogel	Patients were subjected to two HA injections 2 weeks apart. Western Ontario and McMaster Universities Osteoarthritis Index (WOMAC) and Patient’s Global Assessment (PtGA) and Clinical Observer Global Assessment (CoGA) of the disease were assessed at baseline, 30, and 60 days after treatment. *In vivo* human study	This study supports the use of HA in the conservative management of DML as it is clinically effective and enhances meniscus healing as demonstrated by T2 measurements. Moreover, it reduces the need for APM at 1-year follow-up	Berton et al. (2020)
	A injectable alginate dialdehyde-gelatin (15ADA20G) hydrogel	*In vitro* study was performed with rabbit menisci fibrochondrocytes seeded on the hydrogel, and *in vivo* study was conducted in pig meniscal tear model	Adhesion and proliferation of fibrochondrocytes on ADAG hydrogel was confirmed through *in vitro* studies. *Ex vivo* culture on pig meniscus showed integration of hydrogel with meniscal tissue	Resmi et al. (2020)
	TEMPO (2,2,6,6-tetramethylpiperidine-1-oxyl)-oxidized cellulose nanofiber/alginate (TCNF/ALG) hydrogel	Human meniscus fibrochondrocytes (hMFC) from surgical castoffs of partial meniscectomies were mixed with TCNF/ALG precursors and underwent 6 weeks of *in vitro* chondrogenesis in hypoxia prior to analysis with biomechanical, biochemical, molecular, and histological assays	The TCNF/ALG constructs showed more of an inner meniscus-like phenotype while the collagen I-based construct was consistent with a more outer meniscus-like phenotype	Lan et al. (2021)
	Gelatin hydrogel	Mechanical properties were modified with changing gelation PH, and *in vitro* study was conducted to reveal its influences on biochemical and biomechanical properties	Gelation pH is a useful means to modulate both biochemical and biomechanical properties of the collagen-based hydrogels and can be utilized for diverse types of tissue engineering due to its simple application	Kim and Bonassar, (2022)
Artificial hydrogels	Polyvinyl alcohol (PVA)/Na2SO4 hydrogel	Comparative biomechanical analysis was conducted between hydrogel menisci and human donor menisc, and cytotoxicity was evaluated with L929 fibroblasts	PVA/Na2SO4 menisci are mechanically comparable to the human meniscus. Biocompatibility analysis of PVA/Na2SO4 hydrogels revealed no acute cytotoxicity	Hayes et al. (2016)
	Gelatin/polyvinyl alcohol (PVA)-based hydrogel	A mouse fibroblast cell line NIH-3T3 fibroblasts were seeded on the hydrogel, and the integrative capability of the hydrogel was assessed in a meniscal organ-culture model *in vitro*	The combination of gelatin with a PVA-based porous hydrogels allowed to better resemble the mechanical and damping proprieties of native meniscus as well as promoting the integration with the host tissues, as shown by the *ex vivo* test	Marrella et al. (2018)
	Poly (l-glutamic acid) (PLGA)-g-poly (ε-caprolactone) (PCL) hydrogel	The compressive strength was assessed, and the meniscus healing effect was evaluated *in vivo*	The degradation of the PLGA-g-PCL hydrogel was accelerated within 3 months *in vivo*. A hydrogel carrying adipose-derived stem cells (ASCs) effectively regenerated meniscus-like tissue *in vivo* and preserved the corresponding articular cartilage from degeneration over a 16 week period	Zhang et al. (2018a)
	Gelatin methacrylate (GelMA)-agarose (Ag) hydrogel	Human fibrochondrocytes were seeded in agarose (Ag), gelatin methacrylate (GelMA), and GelMA-Ag hydrogels and gene expression were assessed	GelMA-Ag hydrogel was suitable for medial meniscus preparation, and GelMA was conducive to lateral meniscus preparation	Bahcecioglu et al. (2019a)
	Meniscus extracellular matrix (MECM)-poly (ε-caprolactone) (PCL) hydrogel	Rabbit meniscal fibrochondrocytes (MFCs) was used for *in vitro* cell study, and scaffolds were implanted in the meniscus defect *in vivo*	PCL-hydrogel-MFCs group exhibited markedly better gross appearance and cartilage protection than other groups, and the regenerated menisusi in the PCL-hydrogel-MFCs group was similar with native meniscus in the control group	Chen et al. (2019)
	Loofah-chitosan and poly (-3-hydroxybutyrate-co-3-hydroxyvalerate) (PHBV) based hydrogel	Scaffolds were seeded using undifferentiated human mesenchymal stem cells (hMSCs) and incubated for 21 days to investigate the chondrogenic potential of hydrogel scaffolds	The *in vitro* analysis showed no cytotoxic effect and enabled cells to attach, proliferate, and migrate inside the scaffold	Baysan et al. (2022)
	Methacrylate gelatin (GelMA) hydrogels	Mechanical properties and cell viability were examined in the developed hydrogels *in vitro*	GelMA/PEGDMA/HAMA-Hybrid (PGH-Hybrid) had the highest cross-link density, and the developed biomaterials could be used in meniscus tissue engineering with their tunable physicochemical and mechanical properties	Zihna et al. (2022)

## Meniscus regeneration hydrogels

### Cell-based meniscus regeneration hydrogels

For any foreign scaffold, its fusion with native tissue is essential to its functionality. The key to the fusion of hydrogels is the ability of cells to migrate from the normal tissue to the transplanted tissue, and cell-laden hydrogel may accelerate this fusion process. Autologous cells have been shown to play a good role in meniscus substitution (Kon et al., 2012), but their sources are relatively limited. Two papers by Jiang et al. showed that treated pig xenogeneic meniscus tissue could be incorporated with native tissue in the knee joint, but detectable rabbit-anti-pig antibody in the blood serum revealed that the problem of immune rejection requires further study (Jiang et al., 2012; Jiang D. et al., 2018). Compared with autologous cells and xenogenic cells, stem cells may be a more promising cell source in the field of meniscus regeneration research (Zhou et al., 2022). Multiple studies reported that stem cells are useful in replacing the injured meniscus and restoring its native function when combined with tissue engineering (Jacob et al., 2021; Bian et al., 2022). Basing *in vitro* and *in vivo* experiments, Li et al. proved that Apt/GF-scaffolds increased neomeniscal formation in rabbit critical-sized meniscectomies through mesenchymal stem cell (MSC)-specific recruitment (Li H. et al., 2022). Sasaki et al. isolated stem cells from adipose, and demonstrated that adipose-derived stem cells (ASCs)-seeded hydrogels preloaded with TGF-β3 enhanced healing of radial meniscal tears in an *in vitro* meniscal repair model (Sasaki et al., 2018). Zhang et al. suggested that adipose-derived stem cells (ASCs) encapsulated in a biodegradable poly (l-glutamic acid)/poly (ε-caprolactone) hydrogel effectively regenerated meniscus-like tissue *in vivo* and preserved the corresponding articular cartilage from degeneration over a 16 week period (Zhang K. et al., 2018). Hagmeijer et al., investigated the feasibility of a one-stage cell-based treatment for meniscus regeneration by augmenting a resorbable collagen-based implant with a combination of recycled meniscus cells and mesenchymal stromal cells (MSCs), and found that the new one-stage cell-based procedure for meniscus regeneration is feasible, and the stimulatory effect of MSCs towards meniscus cells was regulated by communication through gap junctions (Hagmeijer et al., 2019). Baek et al. examined meniscus tissue generation from different human cell sources including meniscus cells derived from vascular and avascular regions, human bone marrow-derived mesenchymal stem cells, synovial cells, and cells from the infrapatellar fat pad (IPFP), and proved that IPFP cells have potential for use in cell-based meniscus regeneration strategies (Baek et al., 2018). Zhong et al. revealed that rat bone marrow stem cells (BMSCs) mixed with decellularized meniscus extracellular matrix (mECM) hydrogel enabled full-thickness meniscus repair in an orthotopic rat model with *in vitro* 3D cell culture, *in vivo* rat subcutaneous implantation and orthotopic meniscus injury model (Zhong et al., 2020). Ding et al. reviewed a large number of research studies about meniscal repair and regeneration with mesenchymal stem cells from different sources, including bone marrow, peripheral blood, fat, and articular cavity synovium, and determined that current meniscus repair research is still in its infancy, being mostly confined to *in vitro* experiments and animal models. Good healing results may be achieved in animals, but not necessarily in humans. The repair ability of animals is different from that of humans, and meniscus injuries in some animals can be completely healed over a proper period of time without any treatment (Ding G. et al., 2022). Chew et al. analyzed four non-duplicate experiments and concluded that it is not evident that stem cells could repair the meniscus with durable neotissue which is comparable to the original meniscus (Chew et al., 2017). Further clinical trials with standard protocols and long-term follow-ups are required to reveal the influence of stem cells on meniscus regeneration.

### Scaffold-based meniscus regeneration hydrogels

While the search for optimal cell sources is ongoing, some researchers suggested that scaffold-based meniscus regeneration hydrogels may be more important than cells in meniscus regeneration (Chen Y. et al., 2017). Sun et al. found that 3D-bioprinted TCM meniscus not only restored the anisotropy of native healthy meniscus with PBV infiltration and better shape retention, but better maintained joint function and prevented secondary joint degeneration, which demonstrated that the environment of the joint cavity affects meniscus phenotype (Sun et al., 2021). In the meniscus regeneration system, the composition of hydrogels is undoubtedly an important factor affecting cell phenotype changes for the interaction between cells and hydrogels is constant. The ECM scaffold is a native tissue-based strategy for meniscus repair (Zhang Z. et al., 2018). A variety of ECM hydrogels have been reported to be effective in meniscus regeneration (Wu et al., 2015; Yuan et al., 2017; Chen et al., 2019; Zhong et al., 2020; Guo et al., 2021). As the main component of ECM, type I, II and III collagen were all repported to be associated with cultivation of meniscus cells (Mueller et al., 1999; Oda et al., 2015; Wang C. et al., 2020). Reaearch of Mueller et al. showed that type II matrix is beneficial to meniscus regeneration for its resistance to cell-mediated contracture (Mueller et al., 1999), and study of Wang et al. revealed that type III collagen deficiency inhibits meniscal synthesis through mediating the early stage of type II collagen fibrillogenesis and chondrocyte mechanotransduction (Wang C. et al., 2020), but *in vitro* and *in vivo* experiments of Hagmeijer et al. and Oda et al. demonstrated that type I collagen scaffold promotes meniscus regeneration *via* enhancing cellular communication by gap junctions and suppressing inflammation (Oda et al., 2015; Hagmeijer et al., 2019). Tissue-derived scaffolds include ECM scaffolds and other scaffolds prepared from raw materials extracted from biological tissues, such as animal meniscus, small intestinal submucosa, silk, and PRP (Kwon et al., 2019). Scaffolds derived from the menisci of cattle, pigs, rats, and horses were all proven to be effective in meniscus tissue engineering (Yamasaki et al., 2005; Stapleton et al., 2008; Yamasaki et al., 2008; Stapleton et al., 2011; Chen et al., 2015; Visser et al., 2015; Wu et al., 2015; Yuan et al., 2017; Ruprecht et al., 2019; Zhong et al., 2020). As a collagenous biomaterial, small intestinal submucosa was confirmed by many studies to be a capable scaffold for meniscus regeneration (Cook et al., 2001; Gastel et al., 2001; Welch et al., 2002; Cook et al., 2006; Bradley et al., 2007; Tan et al., 2010). Studies by Wu et al. and Yan et al. reported that silk scaffolds coated with platelet-rich gel or collagen can promote functional meniscus regeneration and prevent osteoarthritis (Wu et al., 2019; Yan et al., 2019). PRP is a biological product with scaffolding properties, and it has been suggested to support meniscus healing (Kemmochi et al., 2018; Liu et al., 2019; Kurnaz and Balta, 2020). Natural hydrogel scaffolds derived from sodium alginate, agarose, hyaluronic acid, and gelatin may be the most intensively studied medical biomaterials in the field of tissue engineering. Numerous studies demonstrate that such scaffolds have very good application prospects in the field of meniscus regeneration (Gunja et al., 2009; Echave et al., 2017; Koh et al., 2017; Kremer et al., 2017; Kim et al., 2018; Rastogi and Kandasubramanian, 2019; Berton et al., 2020; Resmi et al., 2020; Abpeikar et al., 2021; Lan et al., 2021; Salahuddin et al., 2021). A study of Bahcecioglu et al. revealed that hydrogels of agarose, and methacrylated gelatin (GelMA) and hyaluronic acid are more supportive for *in vitro* meniscus regeneration than three dimensional printed polycaprolactone scaffolds, and agarose and MeHA could be used for the regeneration of the inner region of meniscus while GelMA for the outer region (Bahcecioglu et al., 2019b). Synthetic hydrogel scaffolds are formed by physical and chemical crosslinking of polymer materials, such as polyvinyl alcohol (PVA), polycaprolactone (PCL), poly (glycolic acid) (PGA), poly-L/DL-lactide (PLDLA), polyethylene glycol (PEG), and polyethylene oxide (PEO) (Burgos-Morales et al., 2021). With advances in technology, standards for the biological activity, mechanical properties, and ease of modification are rising for synthetic materials. In the field of meniscus repair, synthetic materials show good performance (Esposito et al., 2013; Aufderheide and Athanasiou, 2015; Hayes et al., 2016; Marrella et al., 2018; Bahcecioglu et al., 2019a; Cojocaru et al., 2020; Li et al., 2020). Holloway et al. reinforced poly (vinyl alcohol) (PVA) hydrogels with ultrahigh molecular weight polyethylene (UHMWPE) and PP fibers, and suggested that the poly (vinyl alcohol)-based fibrous composite was a possible candidate for meniscal tissue replacement after a series of mechanical evaluations (Holloway et al., 2010). Sthijns et al. reviewed numerous published articles and suggested that synthetic materials can regulate ECM secretions by affecting cellular metabolism and the metabolic state (Sthijns, van Blitterswijk, and LaPointe, 2021), which is promising for the field of biomedical materials. However, since most people assume that synthetic materials may cause unexpected side effects, there is still a long way to go before synthetic materials can be used on a large scale in the human body. Besides, among these four hydrogels (ECM scaffolds, Natrual hydrogel scaffolds, Tissur-derived scaffolds, Synthetic hydrogel scaffolds), which one is more suitable for meniscus regeneration needs to be further confirmed by more studies focusing on the interaction between cells and hydrogels.

### Agent-stimulated meniscus regeneration hydrogels

The human body is an organic whole, but the microenvironment of each local area is unique, differing in parameters such as water content, ion concentration, and pH. Self-assembled polypeptide hydrogels are normally stable and can aggregate spontaneously by forming non-covalent bonds between polypeptide molecules, such as hydrogen bonds, electrostatic interactions, and π-π stacking interactions. Okuno et al. demonstrated that a self-assembling peptide hydrogel scaffold presented a repair and regeneration effect in the meniscus defect of a rabbit model (Okuno et al., 2021).

Non-responsive hydrogels can be classified as traditional hydrogels, and their functions mainly depend on drugs, proteins, and factors loaded on the hydrogels. Research by Zhang et al. suggested that local administration of simvastatin stimulated intrinsic healing of the meniscus (Zhang et al., 2016). Tanaka et al. reported that simvastatin-conjugated gelatin hydrogel could restrain arthritis progression caused by medial meniscectomy (Tanaka et al., 2019). Additionally, PRP-laden hydrogels were shown to exhibit better healing effects than PRP or hydrogel alone in meniscus defects (Ishida et al., 2007; Qi et al., 2021). Other agents loaded on hydrogels also play an important role in regulating the effects of hydrogels. Hydrogels incorporated with transforming growth factor (TGF) were proven to be alternative scaffolds for meniscus healing (Sasaki et al., 2018; Chen et al., 2020). Another growth factor, fibroblast growth factor 2, was indicated to enhance meniscus regeneration with gelatin hydrogel in a rabbit model (Narita et al., 2012).

**FIGURE 3:**
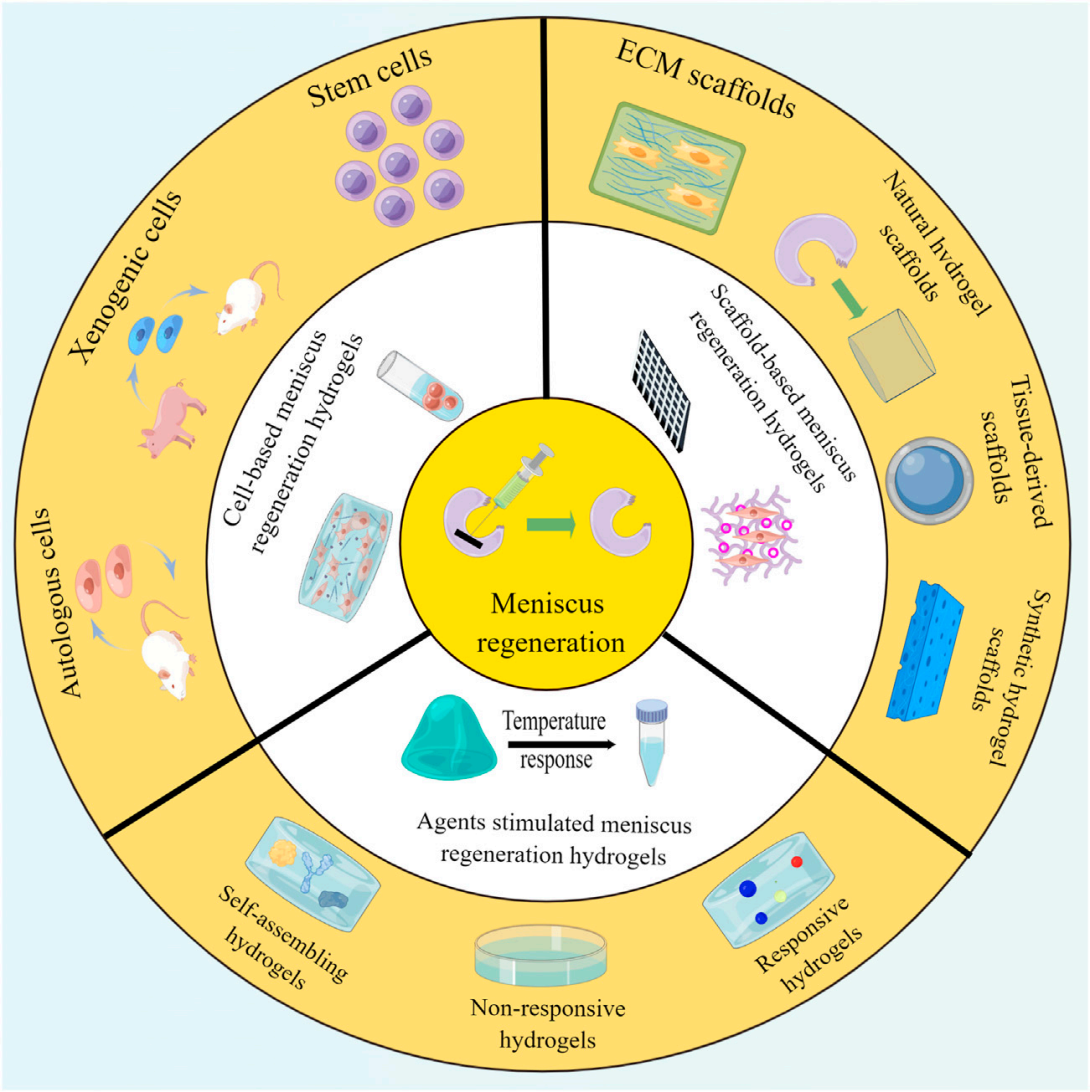
Various hydrogels adopted for meniscus regeneration, including cell-based, scaffold-based, and agent-stimulated meniscus regeneration hydrogels.

Compared with non-responsive hydrogels, stimulus-responsive hydrogels can respond to changes in environmental conditions. Sources of stimulation can be divided into internal stimuli (e.g., pH, redox, enzyme, electricity, and glucose) and external stimuli (e.g., heat, light, mechanical force, magnetic field, and ultrasound). Kim et al. utilized enzyme-based approaches to fabricate tyrosinase (TYR)-crosslinked tissue adhesive hydrogels for meniscus repair and found that these approaches exhibited strong biocompatibility and tissue adhesion (Kim et al., 2018). External stimulus-responsive hydrogels, such as photocrosslinking collagen, photo-curable hydrogel, and thermo-sensitive hydrogel, have been reported as prominent gels for meniscus regeneration (Abpeikar et al., 2021; Karami et al., 2021; Wang et al., 2021). A research by Chen and Cheng found that a thermo-responsive chitosan-graft-poly (N-isopropylacrylamide) injectable hydrogel preserves the viability and phenotypic morphology of chondrocytes and meniscus cells (Chen and Cheng, 2006). Due to the lack of knowledge on the changes in internal factors and signaling pathways after meniscus injury, meniscus repair response hydrogels mainly respond to exogenous stimuli at present. More studies are needed to clarify the underlying mechanisms and, thus, facilitate the creation of more efficient meniscus regeneration hydrogels. Figure 3 and Table 3 exhibit the symbolic experiments of meniscus regeneration hydrogels in meniscus repair and their outcomes.

**TABLE 3:** Summary table of meniscus regeneration hydrogels in meniscus repair and their outcomes.

Classification	Hydrogel	Procedures	Outcomes	References
Cell-based meniscus regeneration hydrogels	Adipose-derived stem cells (ASCs)-seeded hydrogels	Six combinations of hydrogels-namely, acellular and ASC-seeded hydrogels supplemented with preloaded TGF-β3 (2 μg/ml) or soluble TGF-β3 (10 ng/ml) and without supplement-were injected into the radial tear and stabilized by photocrosslinking with visible light. At 4 and 8 weeks of culture, healing was assessed through histology, immunofluorescence staining, and mechanical testing	ASC-seeded hydrogels cultured in medium supplemented with soluble TGF-β3 showed robust proteoglycan deposition. ASC-seeded hydrogels promoted superior healing as compared with acellular hydrogels, with preloaded or soluble TGF-β3 further improving histological scores and mechanical properties	Sasaki et al. (2018)
	A hydrogel carrying adipose-derived stem cells (ASCs)	Poly (l-glutamic acid) (PLGA)-g-poly (ε-caprolactone) (PCL) hydrogel was prepared and the mechanical properties were detected. The regenerative effect on meniscus was assessed *in vivo*	The PLGA-g-PCL hydrogel carrying adipose-derived stem cells (ASCs) effectively regenerated meniscus-like tissue *in vivo* and preserved the corresponding articular cartilage from degeneration over a 16 week period	Zhang et al. (2018a)
	Human meniscus cells (MCs) and mesenchymal stromal cells (MSCs) loaded hydrogel	Human meniscus cells and bone-marrow-derived MSCs were cultured in different ratios in cell pellets and type I collagen hydrogels. In addition, cells were seeded on the implant in fibrin glue by static seeding or injection	The stimulatory effect of MSCs towards meniscus cells was demonstrated by communication through gap junctions	Hagmeijer et al. (2019)
	Rat bone marrow stem cells (BMSCs) delivery hydrogel	The effect of decellularized meniscus extracellular matrix (mECM) on encapsulated MSCs response and integrative meniscus repair by *in vivo* rat subcutaneous implantation and orthotopic meniscus injury model	*In vitro* and *in vivo* researches indicated that mECM hydrogel is a highly promising carrier to deliver MSCs for long-term repair of meniscus tissue	Zhong et al. (2020)
Scaffold-based meniscus regeneration hydrogels	Poly (vinyl alcohol) (PVA) hydrogel	The composite mechanical properties, the molecular weight between cross-links, bound water and the microstructure of the PVA hydrogels were evaluated	The formation of regions with highly concentrated amounts of PVA increases the load-bearing ability of the hydrogels, which may be used as potential non-degradable meniscal replacements	Holloway et al. (2010)
	Porcine meniscus extracellular matrix (MECM) hydrogel	Both bovine chondrocytes and mouse 3T3 fibroblasts were encapsulated in the injectable hydrogel, and was assessed *in vitro* cell culture and *in vivo* mouse subcutaneous implantation	The hydrogel showed good cellular compatibility by promoting the growth of both bovine chondrocytes and mouse 3T3 fibroblasts encapsulated in the hydrogel for 2 weeks. It also promoted cell infiltration as shown in both *in vitro* cell culture and *in vivo* mouse subcutaneous implantation	Wu et al. (2015)
	A salt-modified polyvinyl alcohol hydrogel	Compressive responses were assessed between the hydrogel and human meniscus, and *in vitro* experiments was conducted with L929 fibroblasts	The PVA/Na2SO4 menisci are mechanically comparable to the human meniscus. Biocompatibility analysis of PVA/Na2SO4 hydrogels revealed no acute cytotoxicity	Hayes et al. (2016)
	Decellularized juvenile bovine meniscus ECM hydrogel	Human mesenchymal stem cells (hMSCs) were cutured in mECM hydrogel, and then the hMSC-mECM constructs were assessed with subcutaneous implantation and meniscal injury model in nude rat *in vivo*	The hydrogel was beneficial to fibrochondrogenesis of hMSCs, and enhanced integrative repair of meniscal explantsin orthotopic model	Yuan et al. (2017)
	3D porous gelatin/polyvinyl alcohol (PVA) hydrogel	*In vitro* study was performed with a mouse fibroblast cell line NIH-3T3 fibroblasts, and the implant integration with the host tissue was assessed the *ex vivo* animal model	The combined use of a water-insoluble micro-porogen and gelatin, as a bioactive agent, allowed the realization of a porous composite PVA-based hydrogel to be envisaged as a potential meniscal substitute	Marrella et al. (2018)
	Agarose (Ag), gelatin methacrylate (GelMA), and GelMA-Ag hydrogels	The compressive and tensile modulus and differentiation of the human fibrochondrocytes in these hydrogels were assessed with *in vitro* experiments	GelMA and GelMA-Ag hydrogels enhanced the production of COL 1 and COL 2 proteins after a 6-week culture (*p* < 0.05). COL 1 expression increased gradually towards the outer periphery, while COL 2 expression decreased	Bahcecioglu et al. (2019a)
	Agorose, methacrylated gelatin (GelMA), methacrylated hyaluronic acid (MeHA) and GelMA-MeHA blend hydrogels	Porcine meniscal fibrochondrocytes were cultured in fibrochondrogenic medium, and fibroartilage-related markers were detected to assess its effect *in vitro*	Agarose and MeHA could be used for the regeneration of the inner region of meniscus, while GelMA for the outer region	Bahcecioglu et al. (2019b)
	Poly (ε-caprolactone) (PCL) -meniscus extracellular matrix (MECM)-Based Hydrogel	The efffect of meniscus extracellular matrix (MECM)-based hydrogel in promoting cell proliferation and the phenotype of meniscal fibrochondrocytes (MFCs) and in the knee joints of New Zealand rabbits that underwent total medial meniscectomywas investigated *in vitro* and *in vivo*	The hybrid scaffold (PCL-hydrogel) clearly yielded favorable biomechanical properties close to those of the native meniscus. PCL-hydrogel-MFCs group exhibited markedly better gross appearance and cartilage protection than the PCL scaffold and PCL-hydrogel groups after 6 months postimplantation	Chen et al. (2019)
	Type I collagen hydrogel	Human meniscus cells (MCs) and mesenchymal stem cells (MSCs) were seeded on the implant in fibrin glue by static seeding or injection and meniscus-related assays were performed *in vitro*	20% MCs and 80% MSCs were the most appropriate ratio for a type I collagen hydrogel for meniscus regeneration	Hagmeijer et al. (2019)
	Decellularized meniscus extracellular matrix (mECM) hydrogel	Rat bone marrow stem cells (BMSCs) was cultured in the hydrogel *in vitro*, and evaluated with subcutaneous implantation and meniscus injury modelsin vivo	Injectable ECM hydrogel for delivery of BMSCs enabled full-thickness meniscus repair in an orthotopic rat model	Zhong et al. (2020)
Agent-stimulated meniscus regeneration hydrogels	A thermo-responsive chitosan-graft-poly (N-isopropylacrylamide) injectable hydrogel	A thermo-responsive comb-like polymer with chitosan as the backbone and pendant poly (N-isopropylacrylamide) (PNIPAM) groups has been synthesized by grafting PNIPAM-COOH with a single carboxy end group onto chitosan through amide bond linkages, and assessed with preliminary *in vitro* cell culture study	*In vitro* study demonstrated that the hydrogel not only preserves the viability and phenotypic morphology of the entrapped cells but also stimulates the initial cell-cell interactions, which may be used as an injectable cell-carrier material for entrapping chondrocytes and meniscus cells	Chen and Cheng, (2006)
	Platelet-rich plasma (PRP) loaded gelatin hydrogel	Meniscal cell were cultured in hydrogels, and alcian blue assay and real-time polymerase chain reaction were performed to assess extracellular matrix (ECM) synthesis and the fibrocartilage-related messenger ribonucleic acid (mRNA) expressions. 1.5-mm-diameter full-thickness defects were created, and the defects were filled as follows: Group A, GH with PRP; Group B, GH with platelet-poor plasma; Group C, GH only in the *in vivo* experiments. Each group was evaluated histologically at 4, 8, and 12 weeks after surgery	Histological scoring of the defect sites at 12 weeks revealed significantly better meniscal repair in animals that received PRP with GH than in the other two groups. These findings suggest that PRP enhances the healing of meniscal defects	Ishida et al. (2007)
	Gelatin hydrogels incorporating fibroblast growth factor 2	The *in vivo* effects of gelatin hydrogels (GHs) incorporating fibroblast growth factor 2 (FGF-2) was investigated on meniscus repair in a rabbit model	GHs incorporating FGF-2 significantly stimulated proliferation and inhibited the death of meniscal cells until 4 weeks, thereby increasing meniscal cell density and enhancing meniscal repair in a rabbit model	Narita et al. (2012)
	Gelatin hydrogel and simvastatin-conjugated gelatin hydrogel	In 30 Japanese White rabbits, a cylindrical defect (1.5-mm diameter) was introduced into the avascular zone of the anterior part of the medial meniscus in bilateral knees. Either a gelatin hydrogel (control group) or simvastatin-conjugated gelatin hydrogel (simvastatin group) was implanted into the defect	The qualitative score, mean OR and SOR, immunohistochemical analysis, and biomechanical analysis all demonstrated that simvastatin-conjugated gelatin hydrogel promotes the regeneration of an avascular meniscus in the rabbit model of a meniscal defect and stimulated intrinsic healing of an avascular meniscus	Zhang et al. (2016)
	Tyrosinase (TYR)-crosslinked hydrogels	ECM synthesis and gene expression were assessed with rabbit meniscus fibrochondrocytes *in vitro*	Fibrochondrocyte-laden and TYR-crosslinked hydrogels demonstrated strong biocompatibility and resulted in enhancement of cartilage-specific gene expression and matrix synthesis	Kim et al. (2018)
	A TGF-β3-preloaded photocrosslinkable hydrogel	The effect of TGF-β3-whether preloaded into the hydrogel or added as a soluble medium supplement-on matrix-sulfated proteoglycan deposition in the constructs was evaluated. A meniscal explant culture model was used to simulate meniscal repair	ASC-seeded hydrogels preloaded with TGF-β3 enhanced healing of radial meniscal tears in an *in vitro* meniscal repair model	Sasaki et al. (2018)
	TGF-β1-incorporated hydrogel	Whther the hydrogel could support the fibrochondrogenic differentiation of bone mesenchymal stromal cells (BMSCs) and promote the repair of a critical-sized defect in rabbit meniscus was evaluated *in vitro* and *in vivo*	The hydrogel was biocompatible and could stimulate strong fibrochondrogenic differentiation of BMSCs after the incorporation of TGF-β1. The local administration of the BMSC-laden, TGF-β1-incorporated hydrogel could promote the healing of rabbit meniscal injury	Chen et al. (2020)
	A self-assembling peptide hydrogel scaffold KI24RGDS	Whether the self-assembling peptide hydrogel scaffold KI24RGDS stays in the meniscal lesion and facilitates meniscal repair and regeneration were tested in an induced rabbit meniscal defect model *in vivo*	*In vivo* study demonstrated that KI24RGDS remained in the meniscal lesion and facilitated the repair and regeneration in a rabbit meniscal defect model	Okuno et al. (2021)
	Wnt5a/platelet-rich plasma (PRP) gel	The effect and inflammation reaction of Wnt5a/PRP was investigated on meniscus cells, and the meniscus regeneration and osteoarthritis (OA) prevention was evaluated by the application of Wnt5a/PRP gel in a rabbit model of massive meniscal defect *in vivo*	The IL-1β-induced meniscus cells study showed that PRP and Wnt5a had the anti-inflammatory actions through nuclear factor kB (NF-κB) signaling pathway. PRP and Wnt5a/PRP significantly inhibited the increase of the p-p65/p65 and p-IκB-α/IκB-α ratios. *In vivo* transplantation of Wnt5a/PRP gel was demonstrated to promote meniscus regeneration, while reducing OA of knee joint. Wnt5a with PRP had the anti-inflammatory activity in an IL-1β-induced inflammatory model	Qi et al. (2021)

## Conclusions and outlook

In this review, we presented a discussion on the application of hydrogels to the repair of meniscus damage, organized by the different types and functions of hydrogels, and we described each functionalized hydrogel in detail based on previous studies. Early studies focused on hydrogels for meniscus repair, but with the advancement of material technology and biomedicine, functionalized hydrogels are being developed to achieve the goals of being more bionic and promoting regeneration. Currently, the combination of tissue-derived materials, natural materials, composite materials, nanoparticles, organic polymer materials, and hydrogels has opened up a good future for meniscus repair projects. Meniscus repair will also become more accurate with the help of 3D printing technology.

However, the microenvironment of the meniscus is not static, its connection with surrounding tissue cells will change in different periods, and cells will also remodel ECM components to achieve an environment that is optimal for their existence and function. Intuitively, the repair effect of cell-seeded scaffolds should be better than that of cell-free scaffolds. The study of Numpaisal et al. confirmed that fibrin scaffolds loaded with meniscus cells have better repair effects than scaffolds without cells in radial meniscus tear in hind limbs of young cows (Numpaisal et al., 2017). Basic science researchers are more inclined to reckon that cell-seeded scaffolds are superior than cell-free scaffolds in tissue engineering (Pereira et al., 2011; Kon et al., 2012), and unconsciously pay little attention to convenience and operability of practical application. Two cell-free meniscal scaffolds, collagen-based CMI (Ivy Sports Medicine, Lochhamer, Germany) and polyurethane-based ACTIFIT (Ortheq Bioengineering, Londong, United Kingdom), have been reported to be safe for meniscus replacement, but the neonatal tissue was proved to be different from the native meniscus (Pereira et al., 2019). More high-quality studies are needed to prove the influence of cells on cell-free and cell-seeded scaffolds. With their good biocompatibility, capability for extrinsic substance delivery, and suitability for functional modification, hydrogels are a good choice for exogenous agent delivery and repair of tissue defects. The mechanical property of hydrogel is equally a problem that should be concerned. In order to further detect the regionally biochemical differences of hydrogels from different regions, Wu et al. fabricated regionally decellularized meniscal ECM hydrogels with different (outer, middle, and inner) zones of porcine meniscus, and found that the cell-seeded outer meniscus (OM) hydrogel had a nine-fold increase in peak compressive strengths (18.3 ± 3.6 vs. 2.1 ± 0.1 kPa) and a six-fold increase in initial modulus (9.9 ± 2.6 vs. 1.7 ± 0.2 kPa), the cell-seeded middle meniscus (MM) hydrogel experienced a 21-fold increase in peak compressive strengths (27.1 ± 4.6 vs. 1.3 ± 0.1 kPa) and a nine-fold increase in initial modulus (6.7 ± 1.6 vs. 0.8 ± 0.1 kPa), the cell-seeded inner meniscus (IM) hydrogel achieved a 22-fold increase in peak compressive strengths (26.0 ± 3.0 vs. 1.2 ± 0.2 kPa) and a nine-fold increase in initial modulus (9.4 ± 1.8 vs. 0.9 ± 0.2 kPa) over the IM hydrogel only (Wu et al., 2021). Ding et al. compared the scaffold properties of native meniscus, untreated extracellular matris (ECM) and decellularized ECM (dmECM) from porcine meniscus, and found that both dmECM and untreated ECM scaffolds had lower compressive modulus than native meniscus (181 ± 63 kPa, *p* < 0.001) and the native meniscus was relatively very stiff and showed significantly higher failure compression stresses (2030 ± 250 kPa, *p* < 0.001) (Ding Y. et al., 2022). From these two studies, it can be seen that although biological hydrogels can improve the mechanical strength by adding cells, they are still difficult to achieve the mechanical strength of normal meniscus. If the initial hydrogel is to achieve the mechanical level of normal meniscus, the biohydrogel must be modified. An et al. developed an injectable hydrogel based on fibrin (Fb) reinforced with Pluronic F127 (F127) and polymethyl methacrylate (PMMA), and proved that the gel was beneficial to meniscus repair with the *in vivo* segmental defect of the rabbit meniscus model, but the regenerated tissues of Fb/F127 (3.50 ± 0.35 MPa) and Fb/F127/PMMA (3.59 ± 0.89 MPa) was much higher than that of Fb (0.82 ± 0.05 MPa) but inferior to that of healthy tissue (6.63 ± 1.12 MPa) (An et al., 2021). Kobayashi et al. performed mechanical tests for compression and stress-relaxation among human meniscus and polyvinyl alcohol-hydrogel (PVA-H) with different water content, and detected that the human meniscus has unique viscoelastic properties and compressive strength values of approximately 3 MPa by cutting meniscal samples into small cubes for compression (Kobayashi et al., 2003). However, another report by Beaufils and Versonk demonstrated that human meniscus has a compression value of about 0.15 MPa (Beaufils, 2010). From these studies, we found that the mechanical properties of the meniscus may be different in different species, which may be an important issue to be considered in the future animal experimental research. At the same time, whether the addition of synthetic materials to biological materials will have negative effects on their biological-related properties while improving their mechanical properties, which still needs further study. When considering the mechanical strength of the graft, the poor adhesion between the stent and the surrounding tissue also affects the repair effect. Previous study of Karami et al. prestented a composite double-network hydrogel with a dissipative interface and robustly adheres to soft tissues such as cartilage and meniscus (the adhesion strength is up to 130 kPa) (Karami et al., 2018). The research revealed that chemical properties of hydrogels are easier to improve, but the mechanical strength of meniscus varies from region to region and different regions may have different requirements for adhesive strength of repair materials. Hydrogels that can smartly change adhension strength in different regions should be proposed in the future research.

Overall, this review systematically discussed the applications of different functionalized hydrogels in meniscus repair, focusing on extrinsic substance–delivery hydrogels, meniscus rehabilitation hydrogels, and meniscus regeneration hydrogels. Furthermore, the advantages and disadvantages of different functional hydrogels in meniscus repair were analyzed, and several unsolved problems were highlighted to inspire subsequent research. In summary, hydrogels have good prospects in the application of meniscus repair. Although a variety of hydrogels have been shown to exert positive healing effects in meniscus repair with animal models, there are still few reports of hydrogels achieving regenerative repair in humans. The mechanisms of meniscus development and injury need to be further studied, more multifunctional composite hydrogels that can achieve accurate regeneration of different parts of the meniscus *in situ* and stimulate collagen fibers to grow along the mechanical axis should be proposed, and more conclusive clinical trials should be conducted to screen the effects of these hydrogels.

## References

[B1] Abazari M. Akbari T. Hasani M. Sharifikolouei E. Raoufi M. Foroumadi A. (2022). Polysaccharide-based hydrogels containing herbal extracts for wound healing applications. Carbohydr. Polym. 294, 119808. 10.1016/j.carbpol.2022.119808 35868768

[B2] Abpeikar Z. Javdani M. Mirzaei S. A. Alizadeh A. Moradi L. Soleimannejad M. (2021). Macroporous scaffold surface modified with biological macromolecules and piroxicam-loaded gelatin nanofibers toward meniscus cartilage repair. Int. J. Biol. Macromol. 183, 1327–1345. 10.1016/j.ijbiomac.2021.04.151 33932422

[B3] Akulo K. A. Adali T. Moyo M. T. G. Bodamyali T. (2022). Intravitreal injectable hydrogels for sustained drug delivery in glaucoma treatment and therapy. Polym. (Basel) 14, 2359. 10.3390/polym14122359 PMC9230531 35745935

[B4] An Y. H. Kim J. A. Yim H. G. Han W. J. Park Y. B. Jin P. H. (2021). Meniscus regeneration with injectable Pluronic/PMMA-reinforced fibrin hydrogels in a rabbit segmental meniscectomy model. J. Tissue Eng. 12, 204173142110501. 10.1177/20417314211050141 PMC8552387 34721832

[B5] Aufderheide A. C. Athanasiou K. A. (2015). Comparison of scaffolds and culture conditions for tissue engineering of the knee meniscus. Tissue Eng. 11, 1095–1104. 10.1089/ten.2005.11.1095 16144445

[B6] Baek J. Chen X. Sovani S. Jin S. Grogan S. P. D'Lima D. D. (2015). Meniscus tissue engineering using a novel combination of electrospun scaffolds and human meniscus cells embedded within an extracellular matrix hydrogel. J. Orthop. Res. 33, 572–583. 10.1002/jor.22802 25640671 PMC4386835

[B7] Baek J. Sovani S. Choi W. Jin S. Grogan S. P. Lima D. D. (2018). Meniscal tissue engineering using aligned collagen fibrous scaffolds: Comparison of different human cell sources. Tissue Eng. Part A 24, 81–93. 10.1089/ten.TEA.2016.0205 28463545 PMC5770095

[B8] Bahcecioglu G. Bilgen B. Hasirci N. Hasirci V. (2019a). Anatomical meniscus construct with zone specific biochemical composition and structural organization. Biomaterials 218, 119361. 10.1016/j.biomaterials.2019.119361 31336280

[B9] Bahcecioglu G. Hasirci N. Bilgen B. Hasirci V. (2019b). Hydrogels of agarose, and methacrylated gelatin and hyaluronic acid are more supportive for *in vitro* meniscus regeneration than three dimensional printed polycaprolactone scaffolds. Int. J. Biol. Macromol. 122, 1152–1162. 10.1016/j.ijbiomac.2018.09.065 30218727

[B10] Banovetz M. T. Roethke L. C. Rodriguez A. N. LaPrade R. F. (2022). Meniscal root tears: A decade of research on their relevant anatomy, biomechanics, diagnosis, and treatment. Arch. Bone Jt. Surg. 10, 366–380. 10.22038/abjs.2021.60054.2958 35755791 PMC9194705

[B11] Baysan G. Colpankan Gunes O. Akokay P. Husemoglu R. B. Ertugruloglu P. Ziylan Albayrak A. (2022). Loofah-chitosan and poly (-3-hydroxybutyrate-co-3-hydroxyvalerate) (PHBV) based hydrogel scaffolds for meniscus tissue engineering applications. Int. J. Biol. Macromol. 221, 1171–1183. 10.1016/j.ijbiomac.2022.09.031 36087757

[B12] Beaufils Verdonk (2010). The meniscus. 1st ed. Berlin, Heidelberg, s.l: Springer-Verlag.

[B13] Berton A. Longo U. G. Candela V. Greco F. Martina F. M. Quattrocchi C. C. (2020). Quantitative evaluation of meniscal healing process of degenerative meniscus lesions treated with hyaluronic acid: A clinical and mri study. J. Clin. Med. 9, 2280. 10.3390/jcm9072280 32709084 PMC7408658

[B14] Bian Y. Wang H. Zhao X. Weng X. (2022). Meniscus repair: Up-to-date advances in stem cell-based therapy. Stem Cell Res. Ther. 13, 207. 10.1186/s13287-022-02863-7 35578310 PMC9109379

[B15] Bradley M. P. Fadale P. D. Hulstyn M. J. Muirhead W. R. Lifrak J. T. (2007). Porcine small intestine submucosa for repair of goat meniscal defects. Orthopedics 30, 650–656. 10.3928/01477447-20070801-15 17727022

[B16] Braun H. J. Wasterlain A. S. Dragoo J. L. (2015). The use of PRP in ligament and meniscal healing. Sports Med. Arthrosc. Rev. 21, 206–212. 10.1097/jsa.0000000000000005 24212368

[B17] Burgos-Morales O. Gueye M. Lacombe L. Nowak C. Schmachtenberg R. Hörner M. (2021). Synthetic biology as driver for the biologization of materials sciences. Mat. Today Bio 11, 100115. 10.1016/j.mtbio.2021.100115 PMC8237365 34195591

[B18] Cao Z. Wang H. Chen J. Zhang Y. Mo Q. Zhang P. (2022). Silk-based hydrogel incorporated with metal-organic framework nanozymes for enhanced osteochondral regeneration. Bioact. Mat. 20, 221–242. 10.1016/j.bioactmat.2022.05.025 PMC9163388 35702612

[B19] Chambers H. G. Chambers R. C. (2019). The natural history of meniscus tears. J. Pediatr. Orthop. 39, S53–S55. 10.1097/bpo.0000000000001386 31169650

[B20] Chen C. Song J. Qiu J. Zhao J. (2020). Repair of a meniscal defect in a rabbit model through use of a thermosensitive, injectable, *in situ* crosslinked hydrogel with encapsulated bone mesenchymal stromal cells and transforming growth factor β1. Am. J. Sports Med. 48, 884–894. 10.1177/0363546519898519 31967854

[B21] Chen J. P. Cheng T. H. (2006). Thermo-responsive chitosan-graft-poly(N-isopropylacrylamide) injectable hydrogel for cultivation of chondrocytes and meniscus cells. Macromol. Biosci. 6, 1026–1039. 10.1002/mabi.200600142 17128421

[B22] Chen M. Feng Z. Guo W. Yang D. Gao S. Li Y. (2019). PCL-MECM-Based hydrogel hybrid scaffolds and meniscal fibrochondrocytes promote whole meniscus regeneration in a rabbit meniscectomy model. ACS Appl. Mat. Interfaces 11, 41626–41639. 10.1021/acsami.9b13611 31596568

[B23] Chen S. Fu P. Wu H. Pei M. (2017a). Meniscus, articular cartilage and nucleus pulposus: A comparative review of cartilage-like tissues in anatomy, development and function. Cell Tissue Res. 370, 53–70. 10.1007/s00441-017-2613-0 28413859 PMC5645221

[B24] Chen Y. C. Chen R. N. Jhan H. J. Liu D. Z. Ho H. O. Mao Y. (2015). Development and characterization of acellular extracellular matrix scaffolds from porcine menisci for use in cartilage tissue engineering. Tissue Eng. Part C. Methods 21, 971–986. 10.1089/ten.tec.2015.0036 25919905 PMC4553380

[B25] Chen Y. Chen J. Zhang Z. Lou K. Zhang Q. Wang S. (2017b). Current advances in the development of natural meniscus scaffolds: Innovative approaches to decellularization and recellularization. Cell Tissue Res. 370, 41–52. 10.1007/s00441-017-2605-0 28364144 PMC5610206

[B26] Cheng J. Chen Z. Liu C. Zhong M. Wang S. Sun Y. (2021). Bone mesenchymal stem cell-derived exosome-loaded injectable hydrogel for minimally invasive treatment of spinal cord injury. Nanomedicine (Lond) 16, 1567–1579. 10.2217/nnm-2021-0025 34189939

[B27] Chew E. Prakash R. Khan W. (2017). Mesenchymal stem cells in human meniscal regeneration: A systematic review. Ann. Med. Surg. (Lond). 24, 3–7. 10.1016/j.amsu.2017.09.018 29062478 PMC5644998

[B28] Cojocaru D. G. Hondke S. Krüger J. P. Bosch C. Croicu C. Florescu S. (2020). Meniscus-shaped cell-free polyglycolic acid scaffold for meniscal repair in a sheep model. J. Biomed. Mat. Res. 108, 809–818. 10.1002/jbm.b.34435 31225700

[B29] Cook J. L. Fox D. B. Malaviya P. Tomlinson J. L. Farr J. Kuroki K. (2006). Evaluation of small intestinal submucosa grafts for meniscal regeneration in a clinically relevant posterior meniscectomy model in dogs. J. Knee Surg. 19, 159–167. 10.1055/s-0030-1248100 16893153

[B30] Cook J. L. Tomlinson J. L. Arnoczky S. P. Fox D. B. Reeves Cook C. Kreeger J. M. (2001). Kinetic study of the replacement of porcine small intestinal submucosa grafts and the regeneration of meniscal-like tissue in large avascular meniscal defects in dogs. Tissue Eng. 7, 321–334. 10.1089/10763270152044189 11429152

[B31] Dai W. L. Zhang H. Lin Z. M. Shi Z. J. Wang J. (2019). Efficacy of platelet-rich plasma in arthroscopic repair for discoid lateral meniscus tears. BMC Musculoskelet. Disord. 20, 113. 10.1186/s12891-019-2500-9 30885201 PMC6421692

[B32] Das S. Saha D. Majumdar S. Giri L. (2022). Imaging methods for the assessment of a complex hydrogel as an ocular drug delivery system for glaucoma treatment: Opportunities and challenges in preclinical evaluation. Mol. Pharm. 19, 733–748. 10.1021/acs.molpharmaceut.1c00831 35179892

[B33] Ding G. Du J. Hu X. Ao Y. (2022a). Mesenchymal stem cells from different sources in meniscus repair and regeneration. Front. Bioeng. Biotechnol. 10, 796367. 10.3389/fbioe.2022.796367 35573249 PMC9091333

[B34] Ding Y. Zhang W. Sun B. Mo X. Wu J. (2022b). Cyclic freeze-thaw grinding to decellularize meniscus for fabricating porous, elastic scaffolds. J. Biomed. Mat. Res. A 110, 1824–1839. 10.1002/jbm.a.37435 36082975

[B35] Dos Santos A. M. Carvalho S. G. Araujo V. H. S. Carvalho G. C. Gremião M. P. D. Chorilli M. (2020). Recent advances in hydrogels as strategy for drug delivery intended to vaginal infections. Int. J. Pharm. X. 590, 119867. 10.1016/j.ijpharm.2020.119867 32919001

[B36] Echave M. C. Saenz del Burgo L. Pedraz J. L. Orive G. (2017). Gelatin as biomaterial for tissue engineering. Curr. Pharm. Des. 23, 3567–3584. 10.2174/0929867324666170511123101 28494717

[B37] Esposito A. R. Moda M. Cattani S. M. de Santana G. M. Barbieri J. A. Munhoz M. M. (2013). PLDLA/PCL-T scaffold for meniscus tissue engineering. Biores. Open Access 2, 138–147. 10.1089/biores.2012.0293 23593566 PMC3620496

[B38] Forriol F. Ripalda P. Duart J. Esparza R. Gortazar A. R. (2014). Meniscal repair possibilities using bone morphogenetic protein-7. Injury 45 (4), S15–S21. 10.1016/S0020-1383(14)70005-1 25384469

[B39] Fu X. N. Li H. W. Du N. Liang X. He S. H. Guo K. J. (2020). Erythropoietin enhances meniscal regeneration and prevents osteoarthritis formation in mice. Am. J. Transl. Res. 12, 6464–6477. 33194044 PMC7653595

[B40] Gastel J. A. Muirhead W. R. Lifrak J. T. Fadale P. D. Hulstyn M. J. Labrador D. P. (2001). Meniscal tissue regeneration using a collagenous biomaterial derived from porcine small intestine submucosa. Arthrosc. J. Arthrosc. Relat. Surg. 17, 151–159. 10.1053/jars.2001.20959 11172244

[B41] Gunes O. C. Kara A. Baysan G. Bugra H. R. Akokay P. Ziylan A. A. (2022). Fabrication of 3D Printed poly(lactic acid) strut and wet-electrospun cellulose nano fiber reinforced chitosan-collagen hydrogel composite scaffolds for meniscus tissue engineering. J. Biomater. Appl. 885, 683–697. 10.1177/08853282221109339 35722881

[B42] Gunja N. J. Huey D. J. James R. A. Athanasiou K. A. (2009). Effects of agarose mould compliance and surface roughness on self-assembled meniscus-shaped constructs. J. Tissue Eng. Regen. Med. 3, 521–530. 10.1002/term.191 19658151 PMC2766101

[B43] Guo W. Chen M. Wang Z. Tian Y. Zheng J. Gao S. (2021). 3D-printed cell-free PCL-MECM scaffold with biomimetic micro-structure and micro-environment to enhance *in situ* meniscus regeneration. Bioact. Mat. 6, 3620–3633. 10.1016/j.bioactmat.2021.02.019 PMC8039774 33869902

[B44] Hagmeijer M. H. Vonk L. A. Fenu M. van Keep Y. W. Krych A. J. Saris D. B. (2019). Meniscus regeneration combining meniscus and mesenchymal stromal cells in a degradable meniscus implant: An *in vitro* study. Eur. Cell. Mat. 38, 51–62. 10.22203/ecm.v038a05 31402442

[B45] Hao L. Tianyuan Z. Zhen Y. Fuyang C. Jiang W. Zineng Y. (2021). Biofabrication of cell-free dual drug-releasing biomimetic scaffolds for meniscal regeneration. Biofabrication 14 (1), 015001. 10.1088/1758-5090/ac2cd7 34610586

[B46] Hayes J. C. Curley C. Tierney P. Kennedy J. E. (2016). Biomechanical analysis of a salt-modified polyvinyl alcohol hydrogel for knee meniscus applications, including comparison with human donor samples. J. Mech. Behav. Biomed. Mat. 56, 156–164. 10.1016/j.jmbbm.2015.11.011 26700574

[B47] Heo J. Koh R. H. Shim W. Kim H. D. Yim H. G. Hwang N. S. (2016). Riboflavin-induced photo-crosslinking of collagen hydrogel and its application in meniscus tissue engineering. Drug Deliv. Transl. Res. 6, 148–158. 10.1007/s13346-015-0224-4 25809935

[B48] Herrera Millar V. R. Canciani B. Mangiavini L. Filipe J. F. S. Aidos L. Pallaoro M. (2022). Endostatin in 3D fibrin hydrogel scaffolds promotes chondrogenic differentiation in swine neonatal meniscal cells. Biomedicines 10, 2415. 10.3390/biomedicines10102415 36289678 PMC9598439

[B49] Hoang D. M. Pham P. T. Bach T. Q. Ngo A. T. L. Nguyen Q. T. Phan T. T. K. (2022). Stem cell-based therapy for human diseases. Sig. Transduct. Target. Ther. 7, 272. 10.1038/s41392-022-01134-4 PMC9357075 35933430

[B50] Holloway J. L. Lowman A. M. Palmese G. R. (2010). Mechanical evaluation of poly(vinyl alcohol)-based fibrous composites as biomaterials for meniscal tissue replacement. Acta Biomater. 6, 4716–4724. 10.1016/j.actbio.2010.06.025 20601243

[B51] Ishida K. Kuroda R. Miwa M. Tabata Y. Hokugo A. Kawamoto T. (2007). The regenerative effects of platelet-rich plasma on meniscal cells *in vitro* and its *in vivo* application with biodegradable gelatin hydrogel. Tissue Eng. 13, 1103–1112. 10.1089/ten.2006.0193 17348798

[B52] Jacob G. Shimomura K. Krych A. J. Nakamura N. (2019). The meniscus tear: A review of stem cell therapies. Cells 9, 92. 10.3390/cells9010092 31905968 PMC7016630

[B53] Jacob S. Nair A. B. Shah J. Sreeharsha N. Gupta S. Shinu P. (2021). Emerging role of hydrogels in drug delivery systems, tissue engineering and wound management. Pharmaceutics 13, 357. 10.3390/pharmaceutics13030357 33800402 PMC7999964

[B54] Janarthanan G. Kim J. H. Kim I. Lee C. Chung E. J. Noh I. (2022). Manufacturing of self-standing multi-layered 3D-bioprinted alginate-hyaluronate constructs by controlling the cross-linking mechanisms for tissue engineering applications. Biofabrication 14, 035013. 10.1088/1758-5090/ac6c4c 35504259

[B55] Jansen K. Schuurmans C. C. L. Jansen J. Masereeuw R. Vermonden T. (2017). Hydrogel-based cell therapies for kidney regeneration: Current trends in biofabrication and *in vivo* repair. Curr. Pharm. Des. 23, 3845–3857. 10.2174/1381612823666170710155726 28699526 PMC6302346

[B56] Jiang D. Zhang Z. Z. Zhao F. Wang S. J. Qi Y. S. Zhao L. H. (2018a). The radiated deep-frozen xenogenic meniscal tissue regenerated the total meniscus with chondroprotection. Sci. Rep. 8, 9041. 10.1038/s41598-018-27016-w 29899552 PMC5998046

[B57] Jiang D. Zhao L. H. Tian M. Zhang J. Y. Yu J. K. (2012). Meniscus transplantation using treated xenogeneic meniscal tissue: Viability and chondroprotection study in rabbits. Arthrosc. J. Arthrosc. Relat. Surg. 28, 1147–1159. 10.1016/j.arthro.2012.01.001 22483375

[B58] Jiang G. Li S. Yu K. He B. Hong J. Xu T. (2021). A 3D-printed PRP-GelMA hydrogel promotes osteochondral regeneration through M2 macrophage polarization in a rabbit model. Acta Biomater. 128, 150–162. 10.1016/j.actbio.2021.04.010 33894346

[B59] Jiang T. Wang T. Li T. Ma Y. Shen S. He B. (2018b). Enhanced transdermal drug delivery by transfersome-embedded oligopeptide hydrogel for topical chemotherapy of melanoma. ACS Nano 12, 9693–9701. 10.1021/acsnano.8b03800 30183253

[B60] Karami P. Nasrollahzadeh N. Wyss C. O'Sullivan A. Broome M. Procter P. (2021). An intrinsically-adhesive family of injectable and photo-curable hydrogels with functional physicochemical performance for regenerative medicine. Macromol. Rapid Commun. 42, e2000660. 10.1002/marc.202000660 33834552

[B61] Karami P. Wyss C. S. Khoushabi A. Schmocker A. Broome M. Moser C. (2018). Composite double-network hydrogels to improve adhesion on biological surfaces. ACS Appl. Mat. Interfaces 10, 38692–38699. 10.1021/acsami.8b10735 30335947

[B62] Kemmochi M. Sasaki S. Takahashi M. Nishimura T. Aizawa C. Kikuchi J. (2018). The use of platelet-rich fibrin with platelet-rich plasma support meniscal repair surgery. J. Orthop. 15, 711–720. 10.1016/j.jor.2018.05.006 29881226 PMC5990248

[B63] Kim J. Bonassar L. J. (2022). Controlling collagen gelation pH to enhance biochemical, structural, and biomechanical properties of tissue-engineered menisci. J. Biomed. Mat. Res. A 22, 778. 10.1002/jbm.a.37464 36300869

[B64] Kim S. H. An Y. H. Kim H. D. Kim K. Lee S. H. Yim H. G. (2018). Enzyme-mediated tissue adhesive hydrogels for meniscus repair. Int. J. Biol. Macromol. 110, 479–487. 10.1016/j.ijbiomac.2017.12.053 29229249

[B65] Kobayashi M. Toguchida J. Oka M. (2003). Development of an artificial meniscus using polyvinyl alcohol-hydrogel for early return to, and continuance of, athletic life in sportspersons with severe meniscus injury. I: Mechanical evaluation. Knee 10, 47–51. 10.1016/s0968-0160(02)00152-7 12649026

[B66] Koh R. H. Jin Y. Kang B. J. Hwang N. S. (2017). Chondrogenically primed tonsil-derived mesenchymal stem cells encapsulated in riboflavin-induced photocrosslinking collagen-hyaluronic acid hydrogel for meniscus tissue repairs. Acta Biomater. 53, 318–328. 10.1016/j.actbio.2017.01.081 28161573

[B67] Kon E. Filardo G. Tschon M. Fini M. Giavaresi G. Marchesini Reggiani L. (2012). Tissue engineering for total meniscal substitution: Animal study in sheep model-results at 12 months. Tissue Eng. Part A 18, 1573–1582. 10.1089/ten.tea.2011.0572 22500654

[B68] Kremer A. Ribitsch I. Reboredo J. Dürr J. Egerbacher M. Jenner F. (2017). Three-dimensional coculture of meniscal cells and mesenchymal stem cells in collagen type I hydrogel on a small intestinal matrix-A pilot study toward equine meniscus tissue engineering. Tissue Eng. Part A 23, 390–402. 10.1089/ten.tea.2016.0317 28095754

[B69] Krych A. J. Reardon P. J. Johnson N. R. Mohan R. Peter L. Levy B. A. (2017). Non-operative management of medial meniscus posterior horn root tears is associated with worsening arthritis and poor clinical outcome at 5-year follow-up. Knee Surg. Sports Traumatol. Arthrosc. 25, 383–389. 10.1007/s00167-016-4359-8 27761625

[B70] Kurnaz R. Balta O. (2020). Effect of platelet-rich plasma and platelet-rich fibrin matrix on healing of vertical meniscal tears in a rabbit model. Acta Orthop. Traumatol. Turc. 54, 186–195. 10.5152/j.aott.2020.02.20 32254035 PMC7286167

[B71] Kwon H. Brown W. E. Lee C. A. Wang D. Paschos N. Hu J. C. (2019). Surgical and tissue engineering strategies for articular cartilage and meniscus repair. Nat. Rev. Rheumatol. 15, 550–570. 10.1038/s41584-019-0255-1 31296933 PMC7192556

[B72] Lan X. Ma Z. Szojka A. R. A. Kunze M. Mulet-Sierra A. Vyhlidal M. J. (2021). TEMPO-oxidized cellulose nanofiber-alginate hydrogel as a bioink for human meniscus tissue engineering. Front. Bioeng. Biotechnol. 9, 766399. 10.3389/fbioe.2021.766399 34805119 PMC8602093

[B73] Li H. Zhao T. Cao F. Deng H. He S. Li J. (2022a). Integrated bioactive scaffold with aptamer-targeted stem cell recruitment and growth factor-induced pro-differentiation effects for anisotropic meniscal regeneration. Bioeng. Transl. Med. 7, e10302. 10.1002/btm2.10302 36176622 PMC9472018

[B74] Li S. Dong Q. Peng X. Chen Y. Yang H. Xu W. (2022b2022). Self-healing hyaluronic acid nanocomposite hydrogels with platelet-rich plasma impregnated for skin regeneration. ACS Nano 16, 11346–11359. 10.1021/acsnano.2c05069 35848721

[B75] Li Y. Liu Y. Guo Q. (2021). Silk fibroin hydrogel scaffolds incorporated with chitosan nanoparticles repair articular cartilage defects by regulating TGF-β1 and BMP-2. Arthritis Res. Ther. 23, 50. 10.1186/s13075-020-02382-x 33531052 PMC7856775

[B76] Li Z. Chen Z. Chen H. Chen K. Tao W. Ouyang X. K. (2022c). Polyphenol-based hydrogels: Pyramid evolution from crosslinked structures to biomedical applications and the reverse design. Bioact. Mat. 17, 49–70. 10.1016/j.bioactmat.2022.01.038 PMC8958331 35386465

[B77] Li Z. Wu N. Cheng J. Sun M. Yang P. Zhao F. (2020). Biomechanically, structurally and functionally meticulously tailored polycaprolactone/silk fibroin scaffold for meniscus regeneration. Theranostics 10, 5090–5106. 10.7150/thno.44270 32308770 PMC7163455

[B78] Li Z. Zhao T. Ding J. Gu H. Wang Q. Wang Y. (2022d). A reactive oxygen species-responsive hydrogel encapsulated with bone marrow derived stem cells promotes repair and regeneration of spinal cord injury. Bioact. Mat. 19, 550–568. 10.1016/j.bioactmat.2022.04.029 PMC9108756 35600969

[B79] Liang Y. Idrees E. Szojka A. R. A. Andrews S. H. J. Kunze M. Mulet-Sierra A. (2018). Chondrogenic differentiation of synovial fluid mesenchymal stem cells on human meniscus-derived decellularized matrix requires exogenous growth factors. Acta Biomater. 80, 131–143. 10.1016/j.actbio.2018.09.038 30267878

[B80] Lim H. C. Bae J. H. Wang J. H. Seok C. W. Kim M. K. (2010). Non-operative treatment of degenerative posterior root tear of the medial meniscus. Knee Surg. Sports Traumatol. Arthrosc. 18, 535–539. 10.1007/s00167-009-0891-0 19711053

[B81] Lin D. Lei L. Shi S. Li X. (2019). Stimulus-responsive hydrogel for ophthalmic drug delivery. Macromol. Biosci. 19, e1900001. 10.1002/mabi.201900001 31026123

[B82] Lin J. Wang L. Lin J. Liu Q. (2021). Dual delivery of TGF-β3 and ghrelin in microsphere/hydrogel systems for cartilage regeneration. Molecules 26, 5732. 10.3390/molecules26195732 34641274 PMC8510483

[B83] Liu F. Xu H. Huang H. (2019). A novel kartogenin-platelet-rich plasma gel enhances chondrogenesis of bone marrow mesenchymal stem cells *in vitro* and promotes wounded meniscus healing *in vivo* . Stem Cell Res. Ther. 10, 201. 10.1186/s13287-019-1314-x 31287023 PMC6615105

[B84] Liu Y. Wang M. Luo Y. Liang Q. Yu Y. Chen F. (2021). Enhancing stem cell therapy for cartilage repair in osteoarthritis-A hydrogel focused approach. Gels 7, 263. 10.3390/gels7040263 34940323 PMC8701810

[B85] Liu Z. Tang W. Liu J. Han Y. Yan Q. Dong Y. (2022). A novel sprayable thermosensitive hydrogel coupled with zinc modified metformin promotes the healing of skin wound. Bioact. Mat. 20, 610–626. 10.1016/j.bioactmat.2022.06.008 PMC9256661 35846848

[B86] Longo U. G. Loppini M. Forriol F. Romeo G. Maffulli N. Denaro V. (2012). Advances in meniscal tissue engineering. Stem Cells Int. 2012, 1–7. 10.1155/2012/420346 PMC3205710 25098366

[B87] Lynch C. R. Kondiah P. P. D. Choonara Y. E. du Toit L. C. Ally N. Pillay V. (2020). Hydrogel biomaterials for application in ocular drug delivery. Front. Bioeng. Biotechnol. 8, 228. 10.3389/fbioe.2020.00228 32266248 PMC7099765

[B88] Ma J. Huang C. (2020). Composition and mechanism of three-dimensional hydrogel system in regulating stem cell fate. Tissue Eng. Part B Rev. 26, 498–518. 10.1089/ten.teb.2020.0021 32272868

[B89] Madl C. M. Heilshorn S. C. (2018). Engineering hydrogel microenvironments to recapitulate the stem cell niche. Annu. Rev. Biomed. Eng. 20, 21–47. 10.1146/annurev-bioeng-062117-120954 29220201 PMC7266431

[B90] Maisani M. Sindhu K. R. Fenelon M. Siadous R. Rey S. Mantovani D. (2018). Prolonged delivery of BMP-2 by a non-polymer hydrogel for bone defect regeneration. Drug Deliv. Transl. Res. 8, 178–190. 10.1007/s13346-017-0451-y 29192408

[B91] Marrella A. Lagazzo A. Dellacasa E. Pasquini C. Finocchio E. Barberis F. (2018). 3D porous gelatin/PVA hydrogel as meniscus substitute using alginate micro-particles as porogens. Polym. (Basel) 10, 380. 10.3390/polym10040380 PMC6415243 30966415

[B92] Mills D. K. Luo Y. Elumalai A. Esteve S. Karnik S. Yao S. (2020). Creating structured hydrogel microenvironments for regulating stem cell differentiation. Gels 6, 47. 10.3390/gels6040047 33276682 PMC7768466

[B93] Mueller S. M. Shortkroff S. Schneider T. O. Breinan H. A. Yannas I. V. Spector M. (1999). Meniscus cells seeded in type I and type II collagen-GAG matrices *in vitro* . Biomaterials 20, 701–709. 10.1016/s0142-9612(98)00189-6 10353653

[B94] Nadine S. Correia C. R. Mano J. F. (2022). Engineering immunomodulatory hydrogels and cell-laden systems towards bone regeneration. Biomater. Adv. 140, 213058. 10.1016/j.bioadv.2022.213058 35933955

[B95] Narayanaswamy R. Torchilin V. P. (2019). Hydrogels and their applications in targeted drug delivery. Molecules 24, 603. 10.3390/molecules24030603 30744011 PMC6384686

[B96] Narita A. Takahara M. Sato D. Ogino T. Fukushima S. Kimura Y. (2012). Biodegradable gelatin hydrogels incorporating fibroblast growth factor 2 promote healing of horizontal tears in rabbit meniscus. Arthrosc. J. Arthrosc. Relat. Surg. 28, 255–263. 10.1016/j.arthro.2011.08.294 22119291

[B97] Norouzi M. Nazari B. Miller D. W. (2016). Injectable hydrogel-based drug delivery systems for local cancer therapy. Drug Discov. Today 21, 1835–1849. 10.1016/j.drudis.2016.07.006 27423369

[B98] Numpaisal P. O. Rothrauff B. B. Gottardi R. Chien C. L. Tuan R. S. (2017). Rapidly dissociated autologous meniscus tissue enhances meniscus healing: An *in vitro* study. Connect. Tissue Res. 58, 355–365. 10.1080/03008207.2016.1245727 27726454

[B99] Oda S. Otsuki S. Kurokawa Y. Hoshiyama Y. Nakajima M. Neo M. (2015). A new method for meniscus repair using type I collagen scaffold and infrapatellar fat pad. J. Biomater. Appl. 29, 1439–1448. 10.1177/0885328215568984 25633959

[B100] Okuno N. Otsuki S. Aoyama J. Nakagawa K. Murakami T. Ikeda K. (2021). Feasibility of a self-assembling peptide hydrogel scaffold for meniscal defect: An *in vivo* study in a rabbit model. J. Orthop. Res. 39, 165–176. 10.1002/jor.24841 32852842

[B101] Ozeki N. Muneta T. Koga H. Katagiri H. Otabe K. Okuno M. (2013). Transplantation of Achilles tendon treated with bone morphogenetic protein 7 promotes meniscus regeneration in a rat model of massive meniscal defect. Arthritis Rheum. 65, 2876–2886. 10.1002/art.38099 23897174 PMC4034586

[B102] Pan Z. Wu Y. Zhang X. Fu Q. Li J. Yang Y. (2017). Delivery of epidermal growth factor receptor inhibitor via a customized collagen scaffold promotes meniscal defect regeneration in a rabbit model. Acta Biomater. 62, 210–221. 10.1016/j.actbio.2017.07.008 28757192

[B103] Pereira H. Fatih Cengiz I. Gomes S. Espregueira-Mendes J. Ripoll P. L. Monllau J. C. (2019). Meniscal allograft transplants and new scaffolding techniques. EFORT Open Rev. 4, 279–295. 10.1302/2058-5241.4.180103 31210969 PMC6549113

[B104] Pereira H. Frias A. M. Oliveira J. M. Espregueira-Mendes J. Reis R. L. (2011). Tissue engineering and regenerative medicine strategies in meniscus lesions. Arthrosc. J. Arthrosc. Relat. Surg. 27, 1706–1719. 10.1016/j.arthro.2011.08.283 22019234

[B105] Perinelli D. R. Campana R. Skouras A. Bonacucina G. Cespi M. Mastrotto F. (2018). Chitosan loaded into a hydrogel delivery system as a strategy to treat vaginal Co-infection. Pharmaceutics 10, 23. 10.3390/pharmaceutics10010023 29401648 PMC5874836

[B106] Petersen W. Achtnich A. Lattermann C. Kopf S. (2015). The treatment of non-traumatic meniscus lesions. Dtsch. Arztebl. Int. 112, 705–713. 10.3238/arztebl.2015.0705 26554420 PMC4644934

[B107] Popescu M. B. Carp M. Tevanov I. Nahoi C. A. Stratila M. A. Haram O. M. (2020). Isolated meniscus tears in adolescent patients treated with platelet-rich plasma intra-articular injections: 3-Month clinical outcome. Biomed. Res. Int. 2020, 1–5. 10.1155/2020/8282460 PMC7273443 32596381

[B108] Qi Y. Tang R. Shi Z. Feng G. Zhang W. (2021). Wnt5a/Platelet-rich plasma synergistically inhibits IL-1β-induced inflammatory activity through NF-κB signaling pathway and prevents cartilage damage and promotes meniscus regeneration. J. Tissue Eng. Regen. Med. 15, 612–624. 10.1002/term.3198 33843153

[B109] Qiu M. Chen D. Shen C. Shen J. Zhao H. He Y. (2016). Platelet-rich plasma-loaded poly(d, l-lactide)-Poly(ethylene glycol)-Poly(d, l-lactide) hydrogel dressing promotes full-thickness skin wound healing in a rodent model. Int. J. Mol. Sci. 17, 1001. 10.3390/ijms17071001 27347938 PMC4964377

[B110] Rastogi P. Kandasubramanian B. (2019). Review of alginate-based hydrogel bioprinting for application in tissue engineering. Biofabrication 11, 042001. 10.1088/1758-5090/ab331e 31315105

[B111] Resmi R. Parvathy J. John A. Joseph R. (2020). Injectable self-crosslinking hydrogels for meniscal repair: A study with oxidized alginate and gelatin. Carbohydr. Polym. 234, 115902. 10.1016/j.carbpol.2020.115902 32070521

[B112] Romanazzo S. Vedicherla S. Moran C. Kelly D. J. (2018). Meniscus ECM-functionalised hydrogels containing infrapatellar fat pad-derived stem cells for bioprinting of regionally defined meniscal tissue. J. Tissue Eng. Regen. Med. 12, e1826–e1835. 10.1002/term.2602 29105354

[B113] Ruprecht J. C. Waanders T. D. Rowland C. R. Nishimuta J. F. Glass K. A. Stencel J. (2019). Meniscus-derived matrix scaffolds promote the integrative repair of meniscal defects. Sci. Rep. 9, 8719. 10.1038/s41598-019-44855-3 31213610 PMC6582057

[B114] Salahuddin B. Wang S. Sangian D. Aziz S. Gu Q. (2021). Hybrid gelatin hydrogels in nanomedicine applications. ACS Appl. Bio Mat. 4, 2886–2906. 10.1021/acsabm.0c01630 35014383

[B115] Sasaki H. Rothrauff B. B. Alexander P. G. Lin H. Gottardi R. Fu F. H. (2018). *In vitro* repair of meniscal radial tear with hydrogels seeded with adipose stem cells and TGF-β3. Am. J. Sports Med. 46, 2402–2413. 10.1177/0363546518782973 30001494

[B116] Saygili E. Kaya E. Ilhan-Ayisigi E. Saglam-Metiner P. Alarcin E. Kazan A. (2021). An alginate-poly(acrylamide) hydrogel with TGF-β3 loaded nanoparticles for cartilage repair: Biodegradability, biocompatibility and protein adsorption. Int. J. Biol. Macromol. 172, 381–393. 10.1016/j.ijbiomac.2021.01.069 33476613

[B117] Scotti C. Pozzi A. Mangiavini L. Vitari F. Boschetti F. Domeneghini C. (2009). Healing of meniscal tissue by cellular fibrin glue: An *in vivo* study. Knee Surg. Sports Traumatol. Arthrosc. 17, 645–651. 10.1007/s00167-009-0745-9 19296087

[B118] Shafei S. Khanmohammadi M. Heidari R. Ghanbari H. Taghdiri Nooshabadi V. Farzamfar S. (2020). Exosome loaded alginate hydrogel promotes tissue regeneration in full-thickness skin wounds: An *in vivo* study. J. Biomed. Mat. Res. A 108, 545–556. 10.1002/jbm.a.36835 31702867

[B119] Shao Z. Yin T. Jiang J. He Y. Xiang T. Zhou S. (2022). Wound microenvironment self-adaptive hydrogel with efficient angiogenesis for promoting diabetic wound healing. Bioact. Mat. 20, 561–573. 10.1016/j.bioactmat.2022.06.018 PMC9254353 35846841

[B120] Shen X. Li S. Zhao X. Han J. Chen J. Rao Z. (2022). Dual-crosslinked regenerative hydrogel for sutureless long-term repair of corneal defect. Bioact. Mat. 20, 434–448. 10.1016/j.bioactmat.2022.06.006 PMC9234351 35800407

[B121] Shi W. Fang F. Kong Y. Greer S. E. Kuss M. Liu B. (2021). Dynamic hyaluronic acid hydrogel with covalent linked gelatin as an anti-oxidative bioink for cartilage tissue engineering. Biofabrication 14, 014107. 10.1088/1758-5090/ac42de 34905737

[B122] Shimomura K. Rothrauff B. B. Tuan R. S. (2017). Region-specific effect of the decellularized meniscus extracellular matrix on mesenchymal stem cell-based meniscus tissue engineering. Am. J. Sports Med. 45, 604–611. 10.1177/0363546516674184 27895039

[B123] Simson J. A. Strehin I. A. Allen B. W. Elisseeff J. H. (2013). Bonding and fusion of meniscus fibrocartilage using a novel chondroitin sulfate bone marrow tissue adhesive. Tissue Eng. Part A 19, 1843–1851. 10.1089/ten.TEA.2012.0578 23517453 PMC3700065

[B124] Sonker M. Bajpai S. Khan M. A. Yu X. Tiwary S. K. Shreyash N. (2021). Review of recent advances and their improvement in the effectiveness of hydrogel-based targeted drug delivery: A hope for treating cancer. ACS Appl. Bio Mat. 4, 8080–8109. 10.1021/acsabm.1c00857 35005919

[B125] Stapleton T. W. Ingram J. Fisher J. Ingham E. (2011). Investigation of the regenerative capacity of an acellular porcine medial meniscus for tissue engineering applications. Tissue Eng. Part A 17, 231–242. 10.1089/ten.tea.2009.0807 20695759 PMC3011925

[B126] Stapleton T. W. Ingram J. Katta J. Knight R. Korossis S. Fisher J. (2008). Development and characterization of an acellular porcine medial meniscus for use in tissue engineering. Tissue Eng. Part A 14, 505–518. 10.1089/tea.2007.0233 18370607

[B127] Sthijns M. M. J. P. E. van Blitterswijk C. A. LaPointe V. L. S. (2021). Synthetic materials that affect the extracellular matrix via cellular metabolism and responses to a metabolic state. Front. Bioeng. Biotechnol. 9, 742132. 10.3389/fbioe.2021.742132 34708025 PMC8542861

[B128] Sun Y. Zhang Y. Wu Q. Gao F. Wei Y. Ma Y. (2021). 3D-bioprinting ready-to-implant anisotropic menisci recapitulate healthy meniscus phenotype and prevent secondary joint degeneration. Theranostics 11, 5160–5173. 10.7150/thno.54864 33859740 PMC8039947

[B129] Tan G. K. Cooper-White J. J. (2011). Interactions of meniscal cells with extracellular matrix molecules: Towards the generation of tissue engineered menisci. Cell adh. Migr. 5, 220–226. 10.4161/cam.5.3.14463 21187716 PMC3210205

[B130] Tan Y. Zhang Y. Pei M. (2010). Meniscus reconstruction through coculturing meniscus cells with synovium-derived stem cells on small intestine submucosa-a pilot study to engineer meniscus tissue constructs. Tissue Eng. Part A 16, 67–79. 10.1089/ten.tea.2008.0680 19619075

[B131] Tanaka T. Matsushita T. Nishida K. Takayama K. Nagai K. Araki D. (2019). Attenuation of osteoarthritis progression in mice following intra-articular administration of simvastatin-conjugated gelatin hydrogel. J. Tissue Eng. Regen. Med. 13, 423–432. 10.1002/term.2804 30644168

[B132] Tsou Y. H. Khoneisser J. Huang P. C. Xu X. (2016). Hydrogel as a bioactive material to regulate stem cell fate. Bioact. Mat. 1, 39–55. 10.1016/j.bioactmat.2016.05.001 PMC5883979 29744394

[B133] Tsubosaka M. Kihara S. Hayashi S. Nagata J. Kuwahara T. Fujita M. (2020). Gelatin hydrogels with eicosapentaenoic acid can prevent osteoarthritis progression *in vivo* in a mouse model. J. Orthop. Res. 38, 2157–2169. 10.1002/jor.24688 32270890

[B134] Ulku T. K. Kaya A. Kocaoglu B. (2020). Suture configuration techniques have no effect on mid-term clinical outcomes of arthroscopic meniscus root repairs. Knee 27, 676–682. 10.1016/j.knee.2020.04.017 32563422

[B135] Vernerey F. J. Lalitha Sridhar S. Muralidharan A. Bryant S. J. (2021). Mechanics of 3D cell-hydrogel interactions: Experiments, models, and mechanisms. Chem. Rev. 121, 11085–11148. 10.1021/acs.chemrev.1c00046 34473466

[B136] Visser J. Levett P. A. te Moller N. C. Besems J. Boere K. W. van Rijen M. H. (2015). Crosslinkable hydrogels derived from cartilage, meniscus, and tendon tissue. Tissue Eng. Part A 21, 1195–1206. 10.1089/ten.tea.2014.0362 25557049 PMC4394887

[B137] Wang C. Brisson B. K. Terajima M. Li Q. Hoxha K. Han B. (2020a). Type III collagen is a key regulator of the collagen fibrillar structure and biomechanics of articular cartilage and meniscus. Matrix Biol. 85-86, 47–67. 10.1016/j.matbio.2019.10.001 31655293 PMC7137252

[B138] Wang L. Wang J. Zhou X. Sun J. Zhu B. Duan C. (2020b). A new self-healing hydrogel containing hucMSC-derived exosomes promotes bone regeneration. Front. Bioeng. Biotechnol. 8, 564731. 10.3389/fbioe.2020.564731 33042966 PMC7521201

[B139] Wang Q. S. Xu B. X. Fan K. J. Fan Y. S. Teng H. Wang T. Y. (2021). Dexamethasone-loaded thermo-sensitive hydrogel attenuates osteoarthritis by protecting cartilage and providing effective pain relief. Ann. Transl. Med. 9, 1120. 10.21037/atm-21-684 34430561 PMC8350682

[B140] Waters R. Alam P. Pacelli S. Chakravarti A. R. Ahmed R. P. H. Paul A. (2018). Stem cell-inspired secretome-rich injectable hydrogel to repair injured cardiac tissue. Acta Biomater. 69, 95–106. 10.1016/j.actbio.2017.12.025 29281806 PMC5831493

[B141] Welch J. A. Montgomery R. D. Lenz S. D. Plouhar P. Shelton W. R. (2002). Evaluation of small-intestinal submucosa implants for repair of meniscal defects in dogs. Am. J. Vet. Res. 63, 427–431. 10.2460/ajvr.2002.63.427 11911579

[B142] Wu J. Ding Q. Dutta A. Wang Y. Huang Y. H. Weng H. (2015). An injectable extracellular matrix derived hydrogel for meniscus repair and regeneration. Acta Biomater. 16, 49–59. 10.1016/j.actbio.2015.01.027 25644450

[B143] Wu J. Xu J. Huang Y. Tang L. Hong Y. (2021). Regional-specific meniscal extracellular matrix hydrogels and their effects on cell-matrix interactions of fibrochondrocytes. Biomed. Mat. 17, 014105. 10.1088/1748-605X/ac4178 34883474

[B144] Wu Y. Hong J. Jiang G. Li S. Chen S. Chen W. (2019). Platelet-rich gel-incorporated silk scaffold promotes meniscus regeneration in a rabbit total meniscectomy model. Regen. Med. 14, 753–768. 10.2217/rme-2018-0087 31474179

[B145] Wu Y. Li X. Sun Y. Tan X. Wang C. Wang Z. (2022). Multiscale design of stiffening and ROS scavenging hydrogels for the augmentation of mandibular bone regeneration. Bioact. Mat. 20, 111–125. 10.1016/j.bioactmat.2022.05.021 PMC9133584 35663335

[B146] Xiao Y. Gu Y. Qin L. Chen L. Chen X. Cui W. (2021). Injectable thermosensitive hydrogel-based drug delivery system for local cancer therapy. Colloids Surfaces B Biointerfaces 200, 111581. 10.1016/j.colsurfb.2021.111581 33524696

[B147] Yamasaki T. Deie M. Shinomiya R. Izuta Y. Yasunaga Y. Yanada S. (2005). Meniscal regeneration using tissue engineering with a scaffold derived from a rat meniscus and mesenchymal stromal cells derived from rat bone marrow. J. Biomed. Mat. Res. A 75, 23–30. 10.1002/jbm.a.30369 16049928

[B148] Yamasaki T. Deie M. Shinomiya R. Yasunaga Y. Yanada S. Ochi M. (2008). Transplantation of meniscus regenerated by tissue engineering with a scaffold derived from a rat meniscus and mesenchymal stromal cells derived from rat bone marrow. Artif. Organs 32, 519–524. 10.1111/j.1525-1594.2008.00580.x 18638305

[B149] Yan R. Chen Y. Gu Y. Tang C. Huang J. Hu Y. (2019). A collagen-coated sponge silk scaffold for functional meniscus regeneration. J. Tissue Eng. Regen. Med. 13, 156–173. 10.1002/term.2777 30485706

[B150] Yan W. Xu X. Xu Q. Sun Z. Jiang Q. Shi D. (2022). Platelet-rich plasma combined with injectable hyaluronic acid hydrogel for porcine cartilage regeneration: A 6-month follow-up. Regen. Biomater. 7, 77–90. 10.1093/rb/rbz039 PMC7053269 32153994

[B151] Yang C. P. Hung K. T. Weng C. J. Chen A. C. Hsu K. Y. Chan Y. S. (2021). Clinical outcomes of meniscus repair with or without multiple intra-articular injections of platelet rich plasma after surgery. J. Clin. Med. 10, 2546. 10.3390/jcm10122546 34207554 PMC8228048

[B152] Yazdani M. Shahdadfar A. Jackson C. J. Utheim T. P. (2019). Hyaluronan-based hydrogel scaffolds for limbal stem cell transplantation: A review. Cells 8, 245. 10.3390/cells8030245 30875861 PMC6468750

[B153] Yuan L. Pan M. Lei M. Zhou X. Hu D. Liu Q. (2020). A novel composite of micelles and hydrogel for improving skin delivery of hydrocortisone and application in atopic dermatitis therapy. Appl. Mat. Today 19, 100593. 10.1016/j.apmt.2020.100593

[B154] Yuan T. Li Z. Zhang Y. Shen K. Zhang X. Xie R. (2021). Injectable ultrasonication-induced silk fibroin hydrogel for cartilage repair and regeneration. Tissue Eng. Part A 27, 1213–1224. 10.1089/ten.tea.2020.0323 33353462

[B155] Yuan X. Wei Y. Villasante A. Ng J. J. D. Arkonac D. E. Chao P. G. (2017). Stem cell delivery in tissue-specific hydrogel enabled meniscal repair in an orthotopic rat model. Biomaterials 132, 59–71. 10.1016/j.biomaterials.2017.04.004 28407495 PMC5473162

[B156] Zarrintaj P. Khodadadi Yazdi M. Youssefi Azarfam M. Zare M. Ramsey J. D. Seidi F. (2021). Injectable cell-laden hydrogels for tissue engineering: Recent advances and future opportunities. Tissue Eng. Part A 27, 821–843. 10.1089/ten.tea.2020.0341 33779319

[B157] Zhang F. X. Liu P. Ding W. Meng Q. B. Su D. H. Zhang Q. C. (2021). Injectable Mussel-Inspired highly adhesive hydrogel with exosomes for endogenous cell recruitment and cartilage defect regeneration. Biomaterials 278, 121169. 10.1016/j.biomaterials.2021.121169 34626937

[B158] Zhang K. Wu J. Zhang W. Yan S. Ding J. Chen X. (2018a). *In situ* formation of hydrophobic clusters to enhance mechanical performance of biodegradable poly(l-glutamic acid)/poly(ε-caprolactone) hydrogel towards meniscus tissue engineering. J. Mat. Chem. B 6, 7822–7833. 10.1039/c8tb01453a 32255028

[B159] Zhang L. Li T. Yu Y. Shi K. Bei Z. Qian Y. (2022a). An injectable conductive hydrogel restores electrical transmission at myocardial infarct site to preserve cardiac function and enhance repair. Bioact. Mat. 20, 339–354. 10.1016/j.bioactmat.2022.06.001 PMC9210214 35784639

[B160] Zhang M. Hu W. Cai C. Wu Y. Li J. Dong S. (2022b). Advanced application of stimuli-responsive drug delivery system for inflammatory arthritis treatment. Mat. Today Bio 14, 100223. 10.1016/j.mtbio.2022.100223 PMC8881671 35243298

[B161] Zhang Q. Liu Y. Li J. Wang J. Liu C. (2022c). Recapitulation of growth factor-enriched microenvironment via BMP receptor activating hydrogel. Bioact. Mat. 20, 638–650. 10.1016/j.bioactmat.2022.06.012 PMC9270210 35846838

[B162] Zhang S. Matsushita T. Kuroda R. Nishida K. Matsuzaki T. Matsumoto T. (2016). Local administration of simvastatin stimulates healing of an avascular meniscus in a rabbit model of a meniscal defect. Am. J. Sports Med. 44, 1735–1743. 10.1177/0363546516638342 27159292

[B163] Zhang Z. Guo W. Gao S. Chen M. Li X. Zhang X. (2018b). Native tissue-based strategies for meniscus repair and regeneration. Cell Tissue Res. 373, 337–350. 10.1007/s00441-017-2778-6 29397425

[B164] Zhao P. Feng Y. Zhou Y. Tan C. Liu M. (2022a). Gold@Halloysite nanotubes-chitin composite hydrogel with antibacterial and hemostatic activity for wound healing. Bioact. Mat. 20, 355–367. 10.1016/j.bioactmat.2022.05.035 PMC9207301 35784635

[B165] Zhao Y. P. Han J. F. Zhang F. Y. Liao T. T. Na R. Yuan X. F. (2022b). Flexible nano-liposomes-based transdermal hydrogel for targeted delivery of dexamethasone for rheumatoid arthritis therapy. Drug Deliv. (Lond). 29, 2269–2282. 10.1080/10717544.2022.2096718 PMC9275483 35815790

[B166] Zhong G. Yao J. Huang X. Luo Y. Wang M. Han J. (2020). Injectable ECM hydrogel for delivery of BMSCs enabled full-thickness meniscus repair in an orthotopic rat model. Bioact. Mat. 5, 871–879. 10.1016/j.bioactmat.2020.06.008 PMC7332471 32637750

[B167] Zhou Y. F. Zhang D. Yan W. T. Lian K. Zhang Z. Z. (2022). Meniscus regeneration with multipotent stromal cell therapies. Front. Bioeng. Biotechnol. 10, 796408. 10.3389/fbioe.2022.796408 35237572 PMC8883323

[B168] Zihna G. Topuz B. Günal G. Aydin H. M. (2022). Preparation of hybrid meniscal constructs using hydrogels and acellular matrices. J. Biomater. Sci. Polym. Ed. 22, 1–25. 10.1080/09205063.2022.2135078 36219154

